# Outer kinetochore proteins form linear elements to regulate vesicle transport

**DOI:** 10.1242/jcs.264478

**Published:** 2026-05-11

**Authors:** Shane J. Kowaleski, Alexis Bridgewater, Cody Saraceno, Miranda Dudek, Federico Pelisch, Joshua N. Bembenek

**Affiliations:** ^1^ Department of Obstetrics and Gynecology, C.S. Mott Center for Human Growth and Development, Wayne State University School of Medicine, Detroit, MI 48201, USA; ^2^ Division of Molecular, Cell, and Developmental Biology, School of Life Sciences, University of Dundee, Dundee DD1 5EH, Scotland, UK

**Keywords:** Outer kinetochore, Linear element, Vesicle transport, Oocyte

## Abstract

During cell division, several key regulators of chromosome segregation play additional roles during vesicle trafficking required for cytokinesis. During anaphase I in *Caenorhabditis elegans* oocytes, chromosome segregation is coordinated with vesicle trafficking to support polar body extrusion and exocytosis of extracellular matrix material. Prior to anaphase, numerous outer kinetochore proteins localize to mysterious ‘linear element’ structures throughout the cortex in addition to chromosomes, which has been observed in oocytes of multiple species. Here, we demonstrate that linear elements in *C. elegans* initially form as puncta just before nuclear envelope breakdown and rapidly assemble into larger elongated structures. As linear elements grow, they form large clusters with cortical granule secretory vesicles, initiating an elaborate transport mechanism that distributes vesicles throughout the cortex by anaphase I. Linear elements dynamically interact with microtubules and endoplasmic reticulum during this process. Microtubules are required for linear element assembly, motility and vesicle transport. Knockdown of a plus-end microtubule-binding kinetochore component also inhibits linear element growth and vesicle clustering, but not the motility of linear element puncta. Depletion of several outer kinetochore proteins causes defects in extracellular matrix formation. Therefore, linear elements facilitate the microtubule-dependent transport of vesicles for their proper distribution in the cortex, which is essential for oocyte development.

## Teaser

**Highlighted Article:** Outer kinetochore proteins assemble into cytoplasmic linear elements that facilitate microtubule-dependent vesicle transport during oocyte meiosis, linking chromosome segregation machinery with membrane trafficking.

## INTRODUCTION

The kinetochore is a mechanosensitive microtubule-coupling device and signaling platform required for chromosome segregation during cell division (Kops et al., 2010; Renda and Khodjakov, 2021). Structurally, kinetochores are multilayered assemblies at centromeres, with an outer layer that captures microtubules (Musacchio and Desai, 2017; Walczak et al., 2010). A crucial signaling pathway known as the spindle assembly checkpoint monitors spindle microtubule attachment and tension at kinetochores and inhibits anaphase onset (McAinsh and Kops, 2023; Musacchio and Salmon, 2007). Once proper chromosome alignment is achieved during metaphase, the checkpoint is silenced, allowing the activation of separase (Lara-Gonzalez et al., 2021). Separase is a protease that cleaves cohesin and allows the poleward movement of chromosomes at anaphase onset (Yu et al., 2023).

Separase has multiple functions during anaphase, including the regulation of spindle dynamics (Jensen et al., 2001; Ross and Cohen-Fix, 2004; Sullivan et al., 2001), cyclin-dependent kinase (CDK) activity (Gorr et al., 2005; Yu et al., 2023) and centriole licensing (Matsuo et al., 2012; Tsou et al., 2009). Separase activity is also implicated in the regulation of cytokinesis in several systems (Kudo et al., 2006; Moschou et al., 2013; Stegmeier et al., 2002). We discovered that separase is required for cytokinesis by promoting exocytosis during anaphase (Bembenek et al., 2010). In general, it is thought that cells shut down trafficking during early stages of cell division and resume exocytosis during anaphase to expand the plasma membrane during cytokinesis (Boucrot and Kirchhausen, 2007; Danilchik et al., 2003; Fielding and Royle, 2013; Shuster and Burgess, 2002). A major exocytic pathway involves Rab11-dependent vesicle trafficking from centrosomes to the plasma membrane (Albertson et al., 2005; Riggs et al., 2003; Rothwell et al., 1999; Skop et al., 2001). The regulation of RAB-11 vesicle trafficking by separase allows the cell to stimulate exocytosis in coordination with the onset of chromosome separation at anaphase onset.

During oocyte meiosis I in *Caenorhabditis elegans*, homologous chromosomes are segregated, and one set is partitioned into a small polar body during a highly asymmetric cytokinesis (Fabritius et al., 2011). These events occur during egg activation, a collection of events triggered in oocytes after fertilization (Horner and Wolfner, 2008). Another major egg activation event is the release of cargo through cortical granule exocytosis to modify the extracellular matrix, which provides physical and osmotic protection to the developing embryo (Olson et al., 2012) as well as forming the polyspermy barrier in several species (Liu, 2011). Numerous cell cycle genes have been identified that affect extracellular matrix formation, implicating them in secretion (Sonnichsen et al., 2005; Stein and Golden, 2018). Importantly, both separase and RAB-11 localize to cortical granules and are required for their exocytosis in anaphase I (Bembenek et al., 2007; Sato et al., 2008). This finding implies that the spindle assembly checkpoint pathway, at least through regulation of separase activity, also controls the timing of exocytosis in anaphase. The full extent of cell cycle regulation of vesicle trafficking is still largely unexplored.

Separase localizes to kinetochores and linear elements at early stages of division, and only moves to sites of action at the midbivalent and vesicles at anaphase onset (Bembenek et al., 2007; Sorensen Turpin et al., 2025). Numerous outer kinetochore proteins have also been shown to localize to cytoplasmic linear element structures in *C. elegans* (Dumont et al., 2010; Hattersley et al., 2016, 2022; Howe et al., 2001; Monen et al., 2005; Pelisch et al., 2017; Pereira et al., 2018; Quiogue et al., 2023; Schlientz and Bowerman, 2020; Taylor et al., 2023), *Drosophila* (Gilliland et al., 2007, 2024, 2009) and bovine oocytes (Wu et al., 2023). Although these structures are not well characterized, their colocalization with separase in the cortex implies a potential indirect role in regulating vesicle trafficking. Several interesting observations suggest that outer kinetochore components might have membrane-related functions. The ROD–ZWILCH–ZW10–Spindly (RZZ-S) complex has structural similarity with vesicle coat proteins (Civril et al., 2010; Mosalaganti et al., 2017). ZW10 binds to Syntaxin 18 in an endoplasmic reticulum (ER) tethering complex to regulate ER-Golgi trafficking (Arasaki et al., 2006; Hirose et al., 2004). Multiple kinetochore proteins including Spindly (Moudgil et al., 2015), CENP-E (Ashar et al., 2000; Wu et al., 2024) and CENP-F (Hussein and Taylor, 2002) are farnesylated, a posttranslational modification that can facilitate membrane association (Marshall, 1993). Rab5 regulates chromosome segregation and affects CenpF localization to kinetochores (Serio et al., 2011). ZWINT interacts with Rab3c and SNAP25 and is implicated in vesicle exocytosis (Guo and Yu, 2024; Lee et al., 2002; van Vlijmen et al., 2008). Additionally, the SNARE protein SNAP29 recruits KNL-1 to the kinetochore (Morelli et al., 2016) and co-purifies with Ska1 (Welburn et al., 2009). In addition to these intriguing findings, kinetochore proteins have been shown to have cytoplasmic functions in post-mitotic neurons, providing clear evidence of chromosome-independent functions (Cheerambathur et al., 2019; Del Castillo et al., 2020; Hertzler et al., 2020; Zhao et al., 2019). Therefore, kinetochore proteins have poorly understood cytoplasmic functions and might be involved in various aspects of membrane trafficking.

Here, we demonstrate that linear elements form in *C. elegans* oocytes just before nuclear envelope breakdown (NEBD) from smaller puncta, similar to previous observations in *Drosophila* oocytes (Gilliland et al., 2007). We show that during their formation, linear elements form clusters with cortical granule vesicles during a complex vesicle transport process that ultimately results in vesicle distribution throughout the cortex. Linear elements colocalize with ER and microtubules during their formation. We investigate the function of linear elements during vesicle trafficking and uncover a novel role for outer kinetochore proteins at membranous organelles that occurs concurrently with their well-established function on chromosomes.

## RESULTS

### Linear element dynamics during meiosis I

**Fig. 1.**
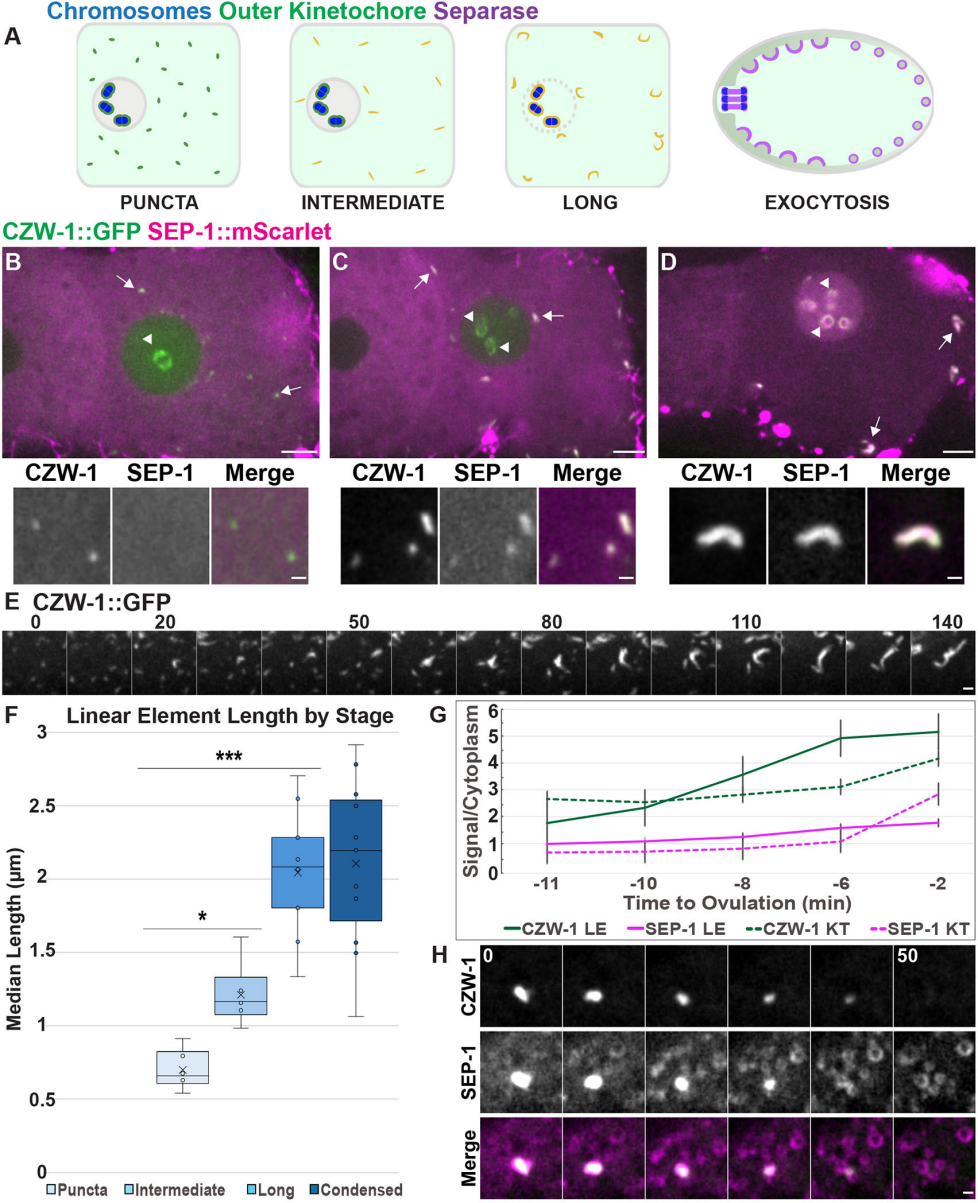
**Linear element dynamics during meiosis I.** (A) Diagrams showing linear elements during *C. elegans* oocyte meiosis I [ZW10 (CZW-1 in worms) is green; separase (SEP-1 in worms) is magenta; colocalization is indicated by yellow]. Initially, small puncta containing only CZW-1 form (left), which rapidly assemble into intermediate and long linear elements containing both CZW-1 and separase (middle). Separase associates with chromosomes (blue) much later than CZW-1. Linear elements disassemble as separase moves to vesicles and promotes exocytosis during anaphase I (right). (B–D) At NEBD, CZW-1::GFP (green) initially appears on small cytoplasmic puncta (B) (arrows) and is already localized to chromosomes (arrowhead), while SEP-1::mScarlet (magenta) remains diffuse in the cytoplasm. (C) SEP-1::mScarlet colocalizes with CZW-1::GFP at linear elements (arrows) when they assemble into intermediate sized structures but is not yet detectable on chromosomes (arrowheads). (D) Just before NEBD, SEP-1::mScarlet colocalizes with CZW-1::GFP on long linear elements and on chromosomes. Fullsize images in B and C are maximum intensity projections, insets are single plane crops of representative linear elements at each stage. Scale bars: 5 µm (main images), 1 µm (magnifications). Images in B–D representative of 6–11 repeats. (E) Montage showing CZW-1::GFP puncta that rapidly assemble into larger structures over the course of several minutes. Images are maximum intensity projections, acquired every 10 s. Scale bar: 1 µm. (F) Linear element lengths were measured from ideal single planes of CZW-1::GFP within the representative time window for puncta (0.70±0.054 µm, *N*=6), intermediate (1.21±0.086 µm, *N*=6), or long (2.04±0.12 µm, *N*=11) (mean±s.e.m.). Puncta are significantly shorter than intermediate (*P=0.025) and long (***P<0.001) (one-way ANOVA followed by post hoc Tukey's test). Condensed stage linear elements were measured across the longest axis in a single focal plane (2.11±0.15 µm, *N*=13). In the graph, each point represents the median length of all linear elements observed in a single plane from one oocyte at the indicated stage. Boxes represent the interquartile range (IQR), with whiskers extending 1.5× the IQR above the third quartile or below the first quartile. The cross marks the mean. (G) Average CZW-1::GFP (green) and SEP-1::mScarlet (magenta) signal intensity ratio over cytoplasm on linear elements (solid lines) and chromosomes (dashed lines) before ovulation (*t*=0). Error bars represent s.e.m. (*N*=6–11). (H) Montage showing CZW-1::GFP (green) and SEP-1::mScarlet (magenta) during anaphase, where CZW-1::GFP disperses and SEP-1 accumulates on cortical granules. Single plane images acquired every 10 s. Images representative of eight repeats. Scale bar: 1 µm.

We recently characterized separase (SEP-1 in *C. elegans*) localization to kinetochores and linear elements and found that it moves to sites of action during anaphase of oocyte meiosis I (Sorensen Turpin et al., 2025). While investigating separase localization, we observed that linear elements form just before NEBD, which marks the start of prometaphase I (*N*=11, Fig. 1A–D). We generated a strain expressing endogenously tagged SEP-1::mScarlet and transgenic CZW-1::GFP, the *C. elegans* ZW10 homolog, to observe linear element dynamics. Initially, small CZW-1::GFP puncta formed and rapidly assembled into longer structures (Fig. 1B,E,G), with SEP-1::mScarlet recruited slightly later (*N*=11, Fig. 1C,G; Movie 1). On average, the separation in time between CZW-1::GFP and SEP-1::mScarlet appearance on linear elements was ∼109±20.87 s (mean±s.e.m.), with CZW-1::GFP first appearing at 605±47.4 s and SEP-1::mScarlet appearing at 496±31.8 s prior to ovulation (*N*=11). Ovulation is defined by the moment when the −1 oocyte detaches from its neighbor and enters the spermatheca. CZW-1::GFP is observed on chromosomes before linear elements form, whereas separase is only recruited to chromosomes after it appears on linear elements (*N*=6, Fig. 1D,G). Therefore, different proteins are recruited to linear elements sequentially, reminiscent of the ordered formation of kinetochores.

Next, we quantified linear element growth over time measuring the length of CZW-1::GFP-labeled structures in single plane images. We divided the process into multiple stages based on size and behavior of linear elements. The initial puncta stage lasted ∼86.90±7.70 s (*N*=5), before most structures are intermediate in size. These intermediate structures were present for ∼93.62±20.18 s (*N*=6) before the initial appearance of longer linear elements is observed. The long linear elements appeared for ∼160.77±29.88 s (*N*=10) before collapsing into consolidated structures that persisted throughout ovulation. The average length of linear elements during these stages increased significantly. CZW-1::GFP puncta had an average length of ∼0.70±0.05 µm (mean±s.e.m.; *N*=6 oocytes, Fig. 1F). Linear elements were significantly longer at the intermediate stage 1.21±0.09 µm (*N*=6) and then reached the long stage with an average length of 2.04±0.12 µm (*N*=11), with some measuring >5 µm. Long linear elements eventually compacted into ‘condensed’ linear elements that measured 2.11±0.15 µm across (*N*=13, Fig. 1F).

Numerous outer kinetochore components and related cell cycle regulators have been reported to associate with linear elements, as summarized in Table S2 (Bai and Bembenek, 2017; Bellutti et al., 2024; Bembenek et al., 2007; Crowder et al., 2015; Dumont et al., 2010; Gigant et al., 2017; Hattersley et al., 2016, 2022; Howe et al., 2001; Monen et al., 2005; Pelisch et al., 2017; Pereira et al., 2018; Quiogue et al., 2023; Schlientz and Bowerman, 2020; Sorensen Turpin et al., 2025; Taylor et al., 2023; Wang et al., 2013; Yang et al., 2026). We verified that numerous outer kinetochore proteins, spindle checkpoint regulators and several microtubule-associated proteins all localized to linear element structures, but not the inner kinetochore protein CENP-C (Fig. S1). To gain insight into what stimulates their formation at NEBD, we RNAi-depleted the cell cycle regulator and linear element component PLK-1 and found linear element formation was significantly inhibited (*N*=11 or 12; Fig. S2). Linear elements persisted in the cortex until mid-anaphase I and were no longer detectable just before SEP-1 localized to cortical granules (*N*=8, Fig. 1G). Therefore, linear elements form in the cortical cytoplasm prior to nuclear envelope breakdown and persist until anaphase when separase moves to vesicles.

### Linear elements assemble into clusters with cortical granules before NEBD

**Fig. 2.**
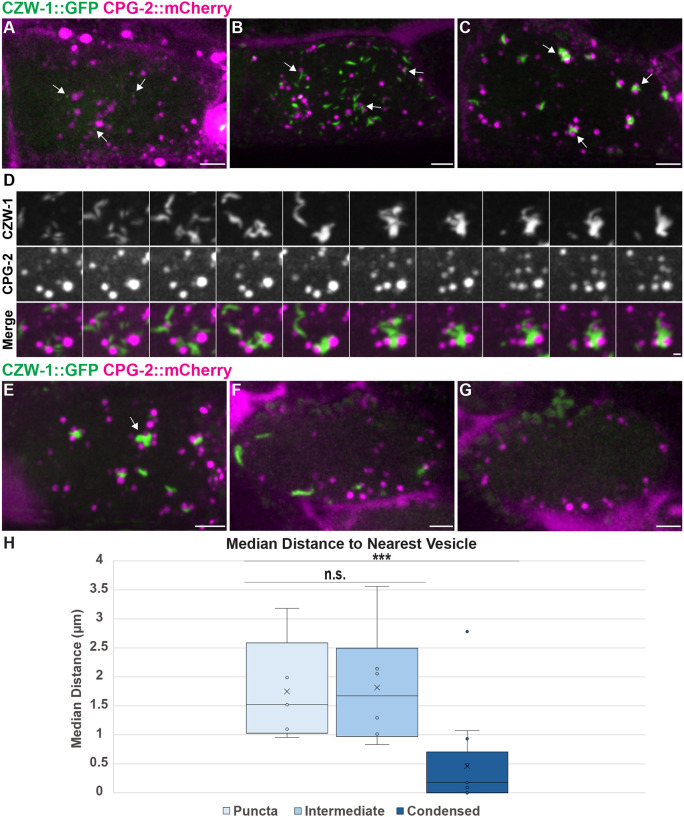
**Linear elements associate with cortical granules.** (A–C) CZW-1::GFP (green, arrows) puncta initially appear in the cortical cytoplasm away from cortical granules marked by CPG-2::mCherry (magenta) (A) but increasingly colocalize with vesicles during their assembly (B) until they are most closely colocalized in clusters just before NEBD (C). (D) Montage showing CZW-1::GFP (green) assembling into a linear element and clustering with CPG-2::mCherry labeled cortical granules (magenta). Images are maximum intensity projections every 30 s. Scale bar: 1 µm. (E,F) Cortical granules (magenta) and linear elements (green) begin to separate as the oocyte moves into the spermatheca (E) and further dissociate during cytoplasmic streaming (F), while linear elements remain relatively stationary. (G) Just before exocytosis in anaphase I, cortical granules (magenta) are distributed throughout the cortex while linear elements disassemble. Images in A–C and E–G are maximum intensity projections. Scale bars: 5 µm. Images in A–G representative of 4–13 repeats. (H) Distance between linear elements and the closest vesicle at different stages of linear element assembly from single plane images. Each point represents the median distances of all linear elements observed in a single plane from one oocyte at the indicated stage. The distance to the nearest vesicle at the condensed stage (0.46±0.22 µm, *N*=13) was significantly closer than the puncta (1.75±0.40 µm, *N*=5, *P*=0.026) or intermediate stages (1.82±0.41 µm, *N*=6, *P*=0.012). ***P<0.001; ns, not significant (one-way ANOVA followed by post hoc Tukey's test). Boxes represent the interquartile range (IQR), with whiskers extending 1.5× the IQR above the third quartile or below the first quartile. The cross marks the mean.

While investigating separase localization to vesicles, we made a striking observation that linear elements colocalized with vesicles at NEBD. We generated a strain expressing CZW-1::GFP and CPG-2::mCherry, a cargo protein of cortical granules, to document linear element and vesicle dynamics. When linear element puncta first formed, they were not closely associated with cortical granules (*N*=5, Fig. 2A,H; Movie 2). As linear elements assembled into larger structures, they dynamically moved in the cortical cytoplasm and increasingly colocalized with cortical granules over time (*N*=6, Fig. 2A–C,H; Movie 2). After linear elements assembled into longer structures, they compacted into clusters with cortical granules at around the time of ovulation (*N*=13, Fig. 2C–E,H; Movie 2). Therefore, linear elements assemble into numerous clusters with cortical granule vesicles at NEBD.

We sought to place linear element clustering with vesicles within the global dynamics of vesicle movements during oocyte meiosis I. Previously, cortical granule vesicles have been observed in close association with the ER, which also undergoes dramatic reorganization (Bembenek et al., 2007; Poteryaev et al., 2005). Therefore, we generated a strain expressing SP12::GFP to visualize the ER, and CPG-2::mCherry to mark cortical granules. During the extensive prophase I arrest that precedes NEBD, oocytes stockpile material necessary for embryonic development. During this ‘production phase’, cortical granules and ER were relatively evenly distributed throughout the cytoplasm (*N*=37, Fig. S3B). Over a 20–30 min period prior to NEBD, the nucleus migrates distally in the oocyte (McCarter et al., 1999). After this period, cortical granules were cortically displaced and the ER was consolidated into thick sheets that were also biased toward the cortex (*N*=27, Fig. S3C). The ER and cortical granules remained cortically displaced through NEBD (*N*=19, Fig. S3D). After ovulation, the oocyte moves into the spermatheca and is fertilized, which occurs during prometaphase I. After fertilization, a dramatic period of cytoplasmic streaming occurs (Kimura et al., 2017). During streaming, the ER became more diffuse and rapidly moved with cortical granules in the cortex (*N*=5, Fig. S3E). Finally, vesicles underwent exocytosis in mid anaphase I (*N*=5, Fig. S3F). Therefore, linear elements cluster with cortical granules in the middle of their journey from early biosynthesis to final fusion with the plasma membrane.

We observed the dynamics of linear elements relative to cortical granules during the different stages of vesicle distribution. Linear elements formed several minutes prior to NEBD, when the ER and cortical granules were already cortically displaced (Fig. 2A; Fig. S3C). As linear elements grow, they cluster with vesicles just before ovulation (*N*=5, Fig. 2E). When oocytes moved into the spermatheca, cortical granules began to dissociate from linear elements (*N*=4, Fig. 2E). Subsequently, cytoplasmic streaming begins and linear elements remained relatively stationary in the cortex while cortical granules moved quickly (Fig. 2F; Movie 3). Finally, linear elements became undetectable during anaphase I just before exocytosis occurred (*N*=4, Fig. 2G), as observed with CZW-1::GFP and several other components (Quiogue et al., 2023; Sorensen Turpin et al., 2025). Together, these observations show that linear elements colocalize with cortical granules around the time of NEBD during a complex vesicle transport process that distributes cortical granules throughout the cortex by anaphase I.

### Linear elements associate with the ER

**Fig. 3.**
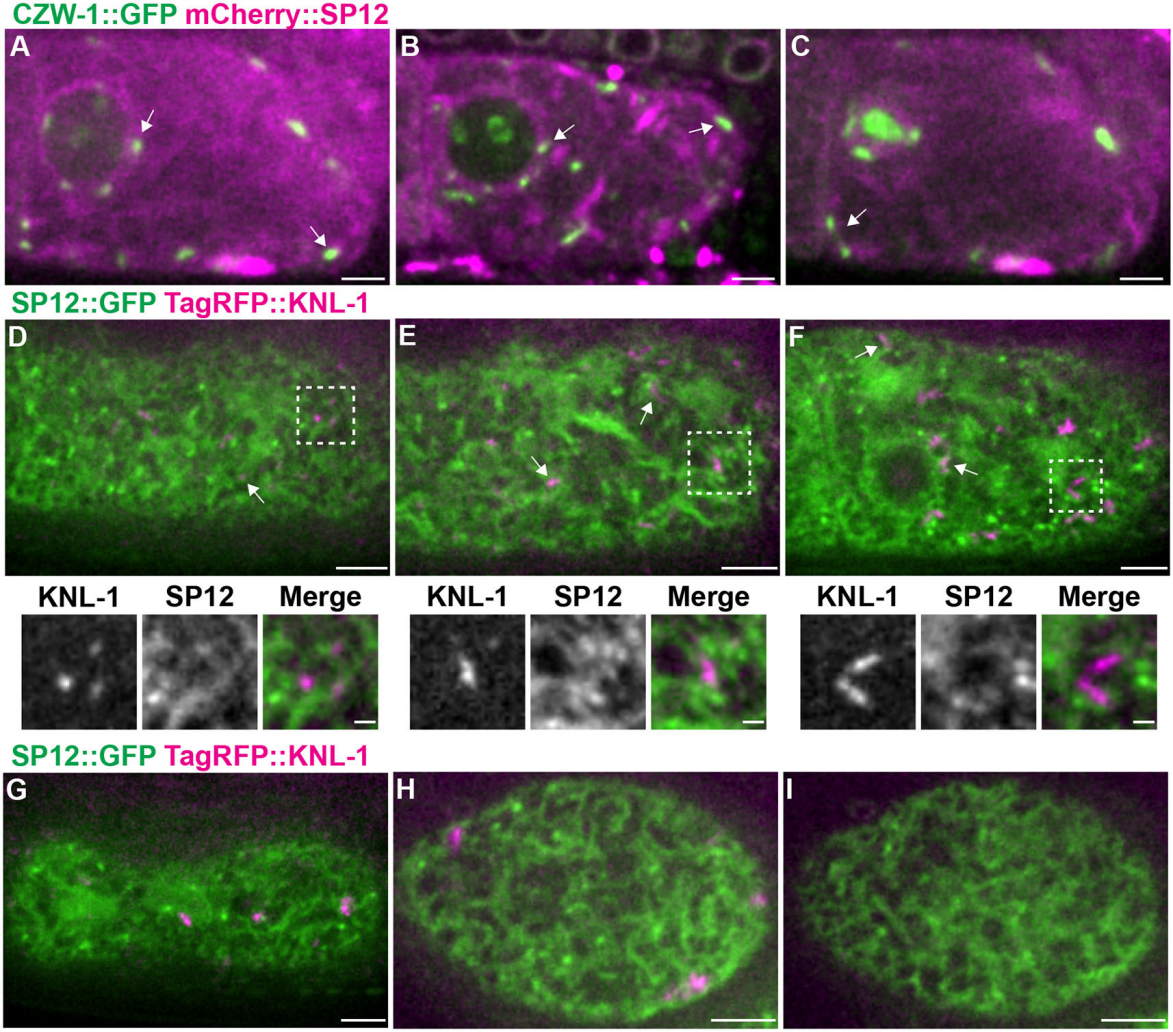
**Linear elements associate with the ER.** (A–C) Nuclear focal planes show that CZW1::GFP (green, arrows) initially forms puncta (A) adjacent to cortical ER and the nuclear envelope marked by mCherry::SP12 (magenta), and they remain associated with the ER and nuclear envelope during the intermediate stage and long stages (B,C). A and C are single plane images, B is a max intensity projection across 1 μm. (D–H) Cortical plane (top surface of the cell) showing TagRFP::KNL-1 puncta (magenta, arrows) (D) within the cortical ER network marked by SP12::GFP (green), which remain embedded within the cortical ER during the intermediate stage (E). Long and condensed linear elements are surrounded by domains of ER just before nuclear envelope breakdown (F) and during ovulation (G). During cytoplasmic streaming, the ER rapidly flows in the cortex, while linear elements are more static and adjacent to ER (H), until their disappearance in anaphase I (I). D–I are single plane images. Images representative of 5–13 repeats. Scale bars: 5 µm (A–C, D–F whole cells, G–I), 1 µm (D–F, magnifications).

As cortical granules interact with the ER throughout meiosis I, we sought to investigate whether linear elements also interact with the ER. We generated strains expressing CZW-1::GFP and mCherry::SP12 and another strain expressing KNL-1 endogenously tagged with TagRFP and SP12::GFP. Linear element puncta associated with ER in the cortex and at the nuclear envelope, which is contiguous with the ER, at the time of their formation (*N*=9, Fig. 3A,D). During their assembly, intermediate sized linear elements closely associated with the ER (*N*=13, Fig. 3B,E). As linear elements increase in size, they associated with ER throughout the cortex and the nuclear envelope through NEBD (*N*=8, Fig. 3C,F; Movie 4), until the time of fertilization (*N*=10, Fig. 3G). During cytoplasmic streaming, linear elements remained static while the ER flowed in the cortex (*N*=5, Fig. 3H; Movie 3). Leading into anaphase I, linear elements became undetectable whereas cortical ER remained distributed throughout the cortex (*N*=5, Fig. 3I). Therefore, linear elements colocalized with the ER from just before NEBD until anaphase I.

### Linear elements interact with the cortical microtubule network

**Fig. 4.**
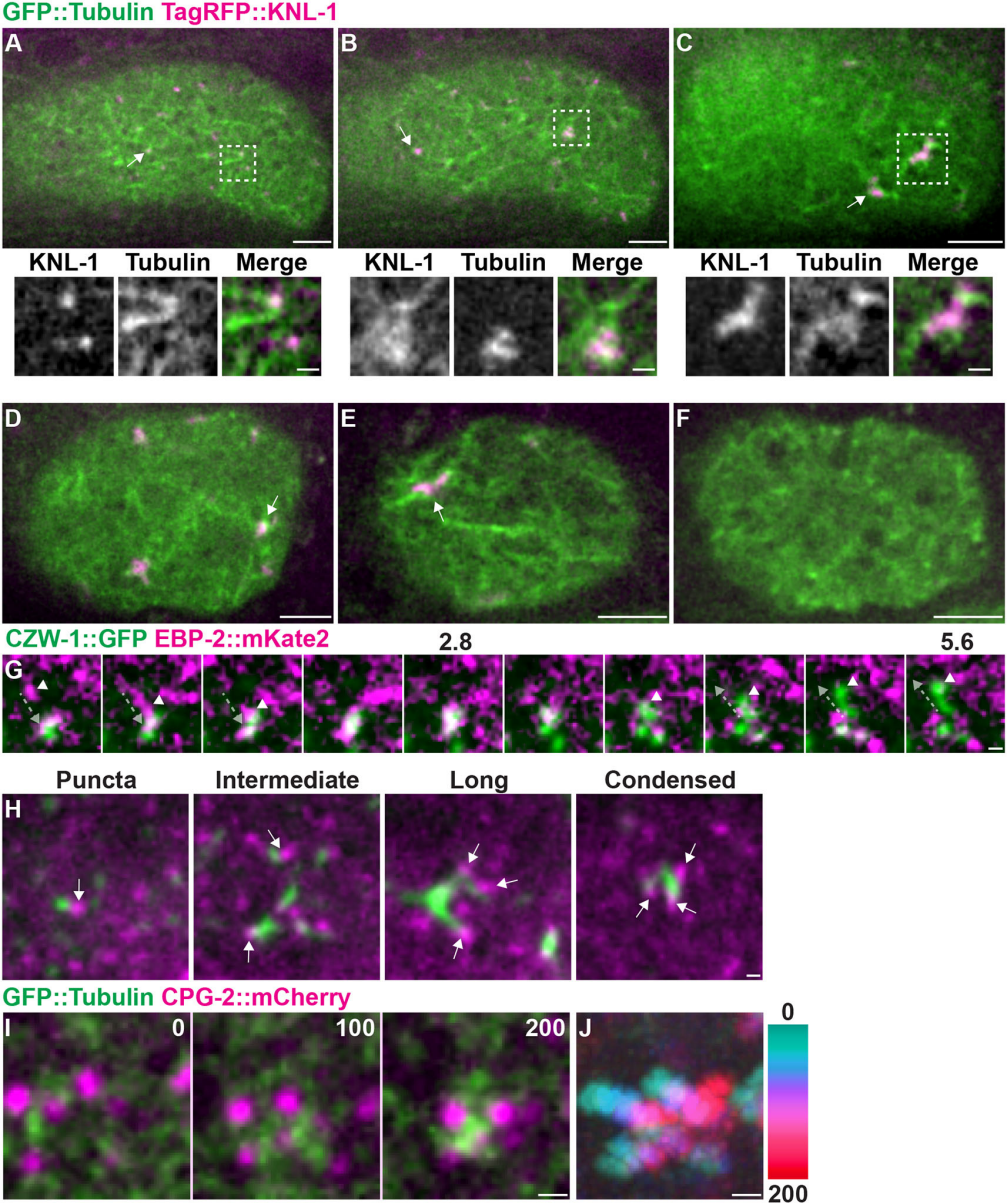
**Linear elements interact with the cortical microtubule network.** (A–F) Linear element puncta (A) marked by TagRFP::KNL-1 (magenta, arrows) appear within the cortical microtubule network (green) and colocalize with accumulations of microtubules at the intermediate and long stages (B,C). Linear elements remain associated with microtubules as the oocyte moves through the spermatheca (D). During cytoplasmic streaming (E), linear elements colocalize with microtubules but remain relatively stationary while microtubules move with cytoplasmic streaming. The cortical microtubule network persists after linear elements have disassembled during anaphase I (F). A–F are single plane images of the top surface of the cell. Scale bars: 5 µm (main images), 1 µm (magnifications). (G) Montage of EBP-2::mKate2 (+ end microtubule marker, magenta) and CZW-1::GFP (green) during linear element assembly. Arrowheads indicate a, EBP-2::mKate2 spot (magenta) moving toward the linear element (left, dashed arrow shows trajectory), and slightly later the linear element (right, arrowheads) moves the reverse direction (dashed arrow). Time interval is 700 ms. Scale bar: 1 µm. (H) EBP-2::mKate2 foci (magenta, arrows), although very dynamic, can be observed in accumulations around linear elements (green) during each stage of linear element assembly. Scale bar: 1 µm. (I) Microtubules (green) accumulate into bundles as cortical granules marked by CPG-2::mCherry (magenta) gather into associated clusters (second and third panels) just prior to fertilization. 100 s separate each panel. Scale bar: 1 µm. (J) Color-coded temporal projection of vesicle clustering (from I), indicating inward movement of vesicles. Images were acquired every 5 s over the course of 200 s. Cyan represents the earliest timepoint (*t*=0), within a gradient turning into red at the last timepoint (*t*=200). Scale bar: 1 μm. Images representative of 4–9 repeats.

The kinetochore mediates spindle microtubule attachment to chromosomes. Therefore, we wanted to investigate the relationship between the linear elements and the cortical microtubule cytoskeleton. We generated a strain expressing endogenously tagged TagRFP::KNL-1 and GFP::tubulin and imaged oocytes during meiosis I. We observed that linear element puncta colocalized with the cortical microtubule network when they first formed (*N*=9, Fig. 4A). As linear elements assembled, they accumulated bundles of microtubules within the network (*N*=6, Fig. 4B). As linear elements elongated, we observed microtubule accumulations at different points along their length (*N*=8, Fig. 4C,D; Movie 5). Linear elements dynamically interacted with microtubules during streaming (*N*=7, Fig. 4E; Movie 3), up until they became undetectable in mid-anaphase I, when cortical microtubule distribution was more even (*N*=5, Fig. 4F). To further investigate the relationship between microtubules and linear elements, we generated a strain containing CZW-1::GFP and EBP-2::mKate2, which labels the plus-end of growing microtubules. EBP-2::mKate2-labeled puncta moved quickly in the cortex. Fast imaging allowed us to observe EBP-2::mKate2-labeled puncta moving towards (Fig. 4G) and away from linear elements, and movement of linear elements sometimes occurred along the same trajectory as EBP-2::mKate2 (Fig. 4G). We observed transient accumulations of EBP-2::mKate2 near linear elements throughout the assembly process (Fig. 4H, *N*=4) Therefore, linear elements colocalize with the cortical microtubule network during meiosis I.

We also investigated the interaction between cortical granules and microtubules using a strain expressing GFP::tubulin and CPG-2::mCherry. Cortical granules localized in the cortical microtubule network prior to NEBD. At NEBD, around the time when linear elements form, cortical granules could be observed packing into clusters that contained a high density of microtubules (*N*=8, Fig. 4I,J). Collectively, these results indicate that linear elements colocalize with microtubules and the ER, and form clusters with cortical granules as they assemble prior to ovulation.

### Kinetochore proteins and microtubules are required for vesicle clustering

**Fig. 5.**
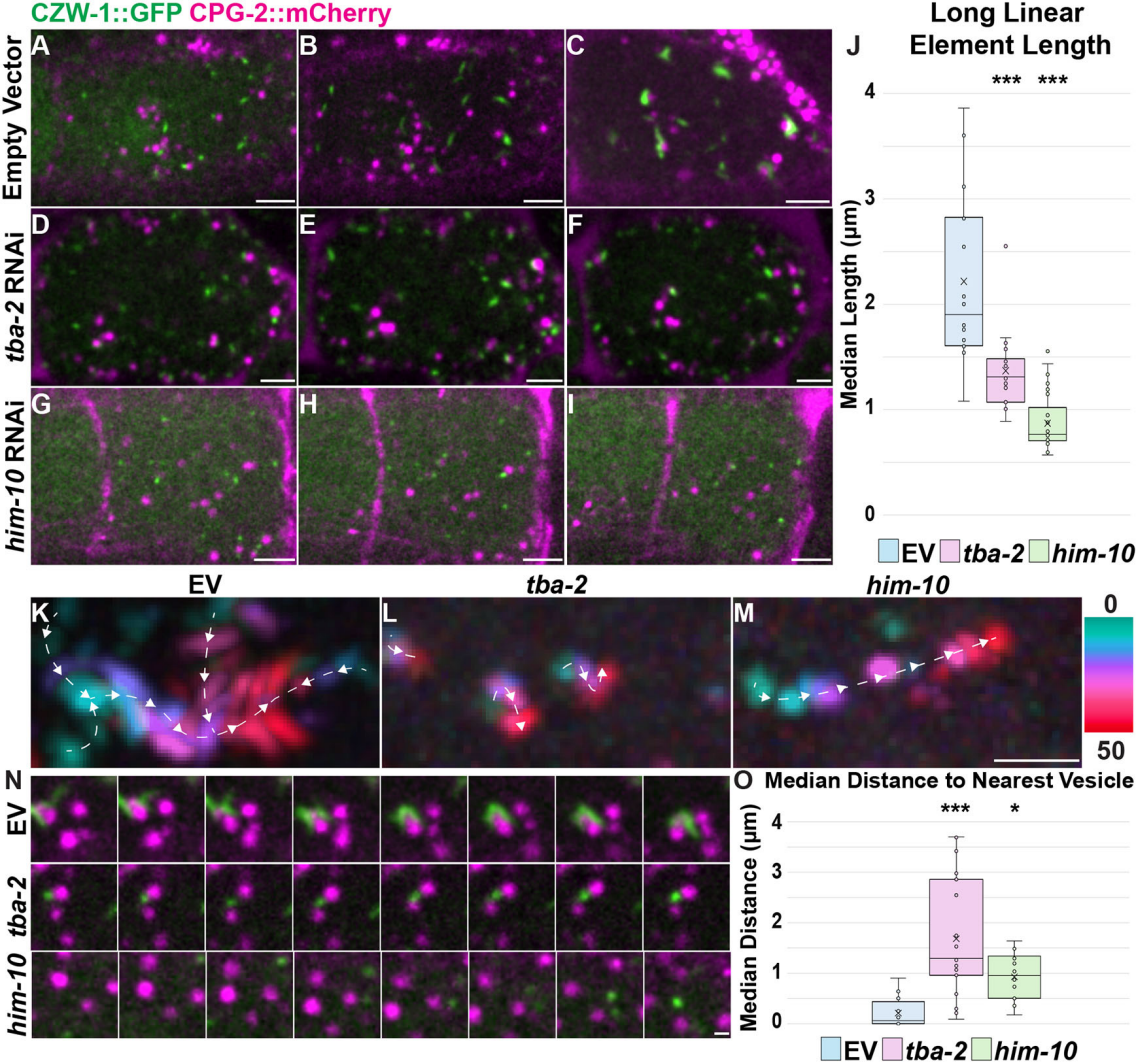
**Microtubules and linear elements are required for vesicle movement.** (A–C) Cortical planes showing CZW-1::GFP forming linear elements (green) and clustering with CPG-2::mCherry-labeled cortical granules (magenta). Puncta (A) assemble into intermediates (B) and eventually form clusters (C) in control oocytes just before NEBD. (D–F) Depletion of microtubules in *tba-2(RNAi)* oocytes severely inhibits linear element (green) assembly and motility. At an early (D), middle (E) or late (F) timepoint, linear elements do not reach full length or form clusters with cortical granules (magenta). (G–I) In *him-10(RNAi)* oocytes, small linear element puncta (green) do not assemble into larger structures at early (G), middle (H), and late (I) timepoints, but are still motile in the cortex. Furthermore, cortical granules (magenta) do not cluster. Scale bars: 5 µm (A–I). (J) Quantification of linear element length in empty vector control, *tba-2(RNAi)* and *him-10(RNAi)* oocytes. Linear element length was measured from a single plane from a time series near ovulation at their longest observable length. Lengths were 2.22±0.20 µm for control (*N*=16), whereas both *tba-2(RNAi)* (1.37±0.083 µm, *N*=19, *P*<0.001) and *him-10 (RNAi)* (0.87±0.049 µm, *N*=30, *P*<0.001) were significantly shorter than control (mean±s.e.m.). (K–M) Color-coded temporal projections of linear elements in control (K), *tba-2(RNAi)* (L), and *him-10(RNAi)* (M) treated oocytes. Linear elements move extensively in control and *him-10(RNAi)* but are relatively stationary after *tba-2(RNAi)* (dashed lines show trajectory of individual objects). Projections represent 50 s in time going from cyan (*t*=0) to red (*t*=+50). Images were acquired every 5 s. Scale bar: 1 μm. (N) Montage of linear element (green) and cortical granule (magenta) behavior in control (empty vector, top), *tba-2(RNAi)* (middle), and *him-10(RNAi)* (bottom) oocytes. Vesicle clustering occurs in control but is inhibited in *tba-2(RNAi)* or *him-10(RNAi)*. Time intervals are 10 s. Scale bar: 1 µm. (O) Quantification of median linear element distance to nearest vesicle. In control (empty vector, *N*=16), the distance was 0.21±0.074 µm, whereas both *tba-2 (RNAi)* (1.68±0.27 µm, *N*=19, *P*<0.001), or *him-10(RNAi)* (0.93±0.14 µm, *N*=12, *P*=0.044) were significantly greater. For plots J and O, each point represents the median of all measurements in a single plane from one oocyte of the indicated condition. Boxes represent the interquartile range (IQR), with whiskers extending 1.5× the IQR above the third quartile or below the first quartile. The cross marks the mean. *P<0.05; ***P<0.001 (one-way ANOVA followed by post hoc Dunnett's test). EV, empty vector.

We next sought to test the hypothesis that linear elements facilitate microtubule-dependent vesicle transport. We investigated linear element and vesicle dynamics at NEBD, using a line expressing CZW-1::GFP and CPG-2::mCherry. To test the role of microtubules in this process, we fed animals *tba-2(RNAi)* for 20–28 h, to generate a long-term depletion of microtubules. Unlike control animals, where linear elements form quickly and cluster with vesicles (Movie 6; Fig. 5A–C,M, top row), linear elements in *tba-2*(*RNAi*) oocytes appeared shorter in length until after NEBD (Fig. 5D–F). We measured linear elements at their longest stage before ovulation and found they were shorter on average in *tba-2(RNAi)* (1.37±0.083 µm, *N*=19; mean±s.e.m.) compared to control (2.22±0.20 µm, *N*=16). After 20–28 h of *tba-2(RNAi)* treatment, we observed mild (*N*=3) to intermediate (*N*=12) to severe (*N*=4) phenotypes. The most severe cases displayed linear elements barely larger than puncta, and vesicle clustering was heavily disrupted (Fig. 5D–F). We measured the distance between linear elements and the closest vesicle and found the average median distance was 1.68±0.27 µm (*N*=19) after *tba-2(RNAi)*, which was significantly greater than the average distance of 0.21±0.07 µm found in control (*P*<0.001, *N*=16, Fig. 5O). Previously, linear element structures have been observed to grow larger after disrupting microtubules (Pereira et al., 2018; Wu et al., 2023). To resolve this apparent discrepancy, we analyzed linear elements after anaphase-promoting complex/cyclosome (APC/C) depletion and found that linear elements could eventually grow larger in long-term prometaphase I-arrested embryos depleted of microtubules (Fig. S4). Linear elements did not cluster with vesicles and were relatively immobile after *tba-2(RNAi)* compared with controls (Fig. 5K,J; Movie 6). Therefore, microtubules are required for efficient linear element motility, assembly and clustering with vesicles.

Additionally, cortical granule distribution appeared abnormal after depleting microtubules prior to linear element formation, indicating an earlier defect in their positioning. To further characterize the role of microtubules in vesicle dynamics, we examined ER and cortical granule distribution after *tba-2(RNAi)* at different stages of meiosis I. In prophase I-arrested *tba-2(RNAi)* oocytes, vesicles remained closer together and the ER was less dispersed throughout the cytoplasm than control (*N*=5, Fig. S3G). Around NEBD, the ER and vesicles were not as cortically displaced in *tba-2(RNAi)* oocytes (*N*=15, Fig. S3H,I) and they remained distant from the cortex through exocytosis in anaphase I (*N*=4, Fig. S3J,K). Crucially, vesicles remained trapped in the cytoplasm after the majority underwent exocytosis in anaphase I (*N*=10 or 11, Fig. S3K). Therefore, microtubule-dependent transport is required for proper cortical granule transport and distribution in the cortex, which is a prerequisite for exocytosis.

To determine whether kinetochore proteins found on linear elements facilitate vesicle movements, we depleted the Nuf2 homolog, *him-10*, an NDC80 complex component involved in plus-end microtubule binding. After 41–46 hours of feeding *him-10(RNAi)*, we observed CZW-1::GFP and CPG-2::mCherry at NEBD. At this timepoint, we observed mild, intermediate and severe phenotypes in *him-10(RNAi)* oocytes based on linear element length before ovulation. Linear element growth was significantly inhibited reaching an average length of 0.87±0.049 µm at the longest point prior to ovulation in *him-10(RNAi)* oocytes (Fig. 5J, *P*<0.001, *N*=30). Linear element clustering with cortical granules was also inhibited before ovulation (Fig. 5G–I,N; Movie 6), with the average median distance to nearest vesicle being 0.929±0.14 µm (*N*=12), approximately four times greater than that of control (0.21±0.074 µm, *N*=16, Fig. 5O, *P*=0.044). Interestingly, linear element puncta in *him-10(RNAi)* oocytes are still motile and move dynamically in the cortex (Fig. 5M). Therefore, Nuf2*^him-10^* is required for linear element assembly and cortical granule clustering but might not be required for motility.

**Fig. 6.**
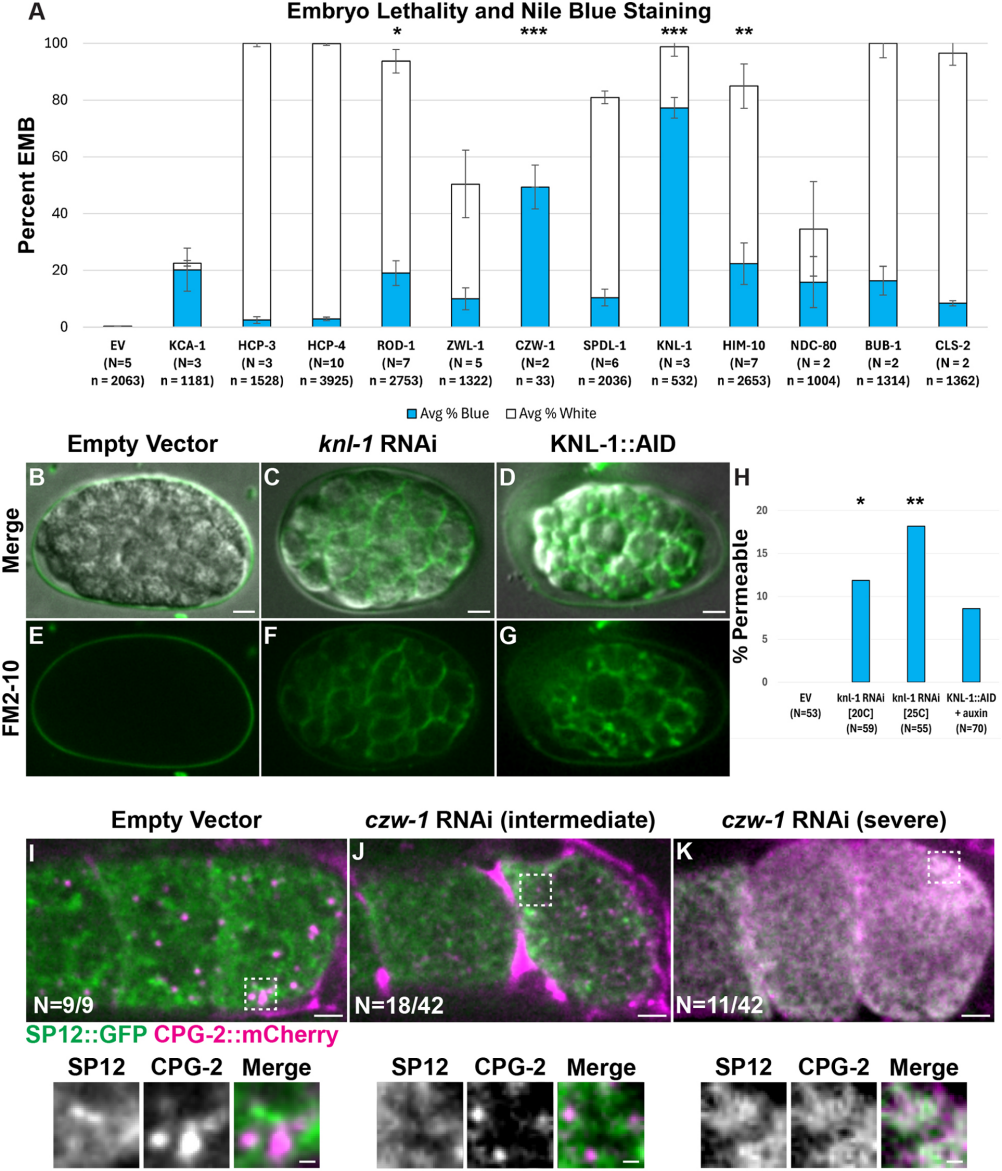
**Outer kinetochore proteins regulate eggshell formation.** (A) RNAi depletion of outer kinetochore proteins causes embryo lethality (total percentage) and eggshell permeability (percentage indicated by blue bars) as assayed by Nile Blue A staining. In comparison, depletion of the kinesin motor adapter, KCA-1, causes permeability as previously shown (Carvalho et al., 2011; Olson et al., 2012). Depletion of several outer kinetochore proteins causes eggshell permeability, more than depletion of centromeric (CENP-A; HCP-3 in worms) and inner kinetochore (CENP-C; HCP-4 in worms) proteins. Error bars indicate s.e.m. *N* indicates the number of experimental replicates for each condition, *n* is number of embryos counted. *P<0.05; **P<0.01; ***P<0.001 (one-way ANOVA followed by a post hoc Dunnett's test). (B–G) Representative images of embryos dissected into FM2-10 from N2 adults treated with empty vector RNAi (B,E), *knl-1* RNAi (C,F), or endogenously tagged AID::KNL-1 adults exposed to auxin (D,G). Scale bars: 5 µm. (H) The total fraction of embryos permeable to FM2-10 in control or *knl-1* knockdown conditions. The percentage of stained embryos observed for each experiment treated with control (*N*=3 experiments for a total of 53 embryos examined), *knl-1(RNAi)* at 20°C (*N*=3 experiments for 59 embryos), *knl-1(RNAi)* at 25°C (*N*=2 experiments for 55 embryos), or KNL-1::AID+auxin (*N*=4 experiments for 70 embryos) were compared. Significantly higher staining was observed in worms treated with *knl-1(RNAi)* grown at 20°C (*P*=0.047) or 25°C (*P*=0.0088). *P<0.05; **P<0.01 (one-way ANOVA followed by a post hoc Dunnett's test). EV, empty vector. (I–K) Representative images of ER (SP12::GFP, green) and cortical granule (CPG-2::mCherry, magenta) distribution in the germline of worms fed control (empty vector) RNAi (I) or *czw-1(RNAi)* (J,K). After 24–25 h of feeding, we observed an intermediate *czw-1(RNAi)* phenotype in 18/42 cases where CPG-2::mCherry appears in smaller puncta (J), whereas in severe cases (K, 11/42 animals imaged), most CPG-2::mCherry signal remains trapped in the ER. Scale bars: 5 µm (main images), 1 µm (magnifications).

Cortical granule exocytosis is required for eggshell formation. Therefore, we performed a small-scale RNAi screen using Nile Blue plates to detect permeable embryos, an indication that eggshell formation is disrupted. Depletion of numerous outer kinetochore proteins causes embryo lethality, with a fraction that showed a significant increase in Nile Blue staining (Fig. 6A). We dissected embryos into buffer containing the membrane dye FM2-10. In control embryos, FM2-10 did not label internal membranes after embryos began the mitotic divisions, indicating that the eggshell is impermeable (Fig. 6B,E,H). In contrast, embryos depleted of KNL-1 by RNAi or auxin-induced degradation had internal membrane staining with FM2-10 (Fig. 6C,D,F–H). Finally, we depleted CZW-1, which was previously categorized as causing the osmotic integrity defective phenotype (Sonnichsen et al., 2005). ER and cortical granules were found normally in control oocytes, whereas *czw-1(RNAi)* inhibited the packaging of CPG-2::Cherry into vesicles and instead it localized to the ER (Fig. 6I,K). Therefore, kinetochore proteins support an elaborate vesicle transport process necessary for efficient cargo exocytosis during anaphase I and subsequent eggshell formation.

## DISCUSSION

Many outer kinetochore proteins have been observed in linear elements since Nuf2^him-10^ localization was first reported in oocyte meiosis I in *C. elegans* (Howe et al., 2001). Their function has remained enigmatic given that kinetochore proteins are primarily understood for their function at chromosomes, not in the cytoplasm. Linear elements share many properties with the fibrous corona, an expansion of the kinetochore that facilitates microtubule capture early in mitosis (Kops and Gassmann, 2020). While linear elements have been observed primarily in oocytes, kinetochore proteins were previously observed in ‘flares’ extending from chromosomes in mitotic cells under checkpoint activating conditions (Stear and Roth, 2004). In addition, ROD-1 can form filaments in mitotic cells under certain conditions (Pereira et al., 2018). Our finding that linear elements interact with the ER and vesicles and facilitate microtubule mediated vesicle movement is a newly discovered function for these structures. Linear element function is both temporally and spatially distinct from kinetochores given that they form in the cortical cytoplasm and colocalize with membranes before NEBD, when kinetochores first interact with spindle microtubules. These results might explain why some kinetochore proteins have interactions with membrane trafficking proteins.

The role of linear elements in vesicle transport appears to be part of an elaborate process that globally organizes the oocyte cytoplasm. We define several distinct stages of ER and vesicle organization from prophase I arrest through anaphase I, when exocytosis occurs. Linear element formation occurs after the nucleus moves distally and the ER and vesicles are cortically displaced. Microtubules and kinesin activity are required for distal nuclear positioning (McNally et al., 2010; Yang et al., 2003) and we find that microtubules regulate ER and vesicle distribution as well. In mouse oocytes, vesicle movement to the cortex is mediated by actin (Schuh, 2011), which could also be involved in *C. elegans*. The vesicle clustering event involving linear elements occurs just prior to NEBD. Interestingly, spindle checkpoint components function at the nuclear envelope to time NEBD (Cunha-Silva and Conde, 2020), which could also regulate linear elements. Indeed, spindle checkpoint and nuclear pore components localize to linear elements (Hattersley et al., 2022). Following the clustering event, a dynamic process of cytoplasmic streaming occurs, which has been observed in multiple cell types (Lu and Gelfand, 2023). Previously, the ER and microtubules have been shown to be required for streaming, which distributes cortical granules in the cortex for efficient exocytosis in anaphase I (Kimura et al., 2017). The role of linear elements during streaming is not clear, but they remain relatively stationary in the cortex whereas the ER, vesicles and microtubules flow with the cytoplasm. Interestingly, depletion of CLS-2, which is observed at linear elements, disrupts the cortical microtubule network and affects polar body extrusion (Quiogue et al., 2023). Finally, kinesin packs organelles inward to facilitate spindle positioning (Aquino et al., 2025; McNally et al., 2010). Depletion of the KCA-1 kinesin adapter has previously been shown to cause vesicles to be retained in meiosis II, suggesting a role in cortical granule transport (Olson et al., 2012). The fact that multiple mechanisms govern cytoplasmic organization in the oocyte might explain why depletion of linear element kinetochore proteins does not cause a severe vesicle exocytosis defect. Additionally, we found that RNAi depletion for over 40-h is less efficient than auxin-induced degradation of KNL-1 (animals go sterile in under 20 h), indicating that our depletions might not be fully penetrant. In addition, remaining kinetochore subcomplexes might have sufficient activity to move vesicles to the cortex. Multiple mechanisms could be redundant or act in parallel with linear elements, enhancing the efficiency of the process. Future studies will be required to fully understand the role of linear elements during vesicle transport in the oocyte.

One important question is what mechanism(s) are required for linear element assembly. We observe small puncta rapidly increasing in size to intermediate structures that appear to merge and build longer structures. This pattern is reminiscent of cytoophidia, structures made of polymerized CTP synthase, that start as small puncta that fuse into similar elongated filamentous structures (Liu, 2016). This suggests a stepwise formation process with a hierarchy of assembly, as is the case for kinetochore formation on chromosomes (Navarro and Cheeseman, 2021). Indeed, recruitment of KNL-1, CLS-2 and BUB-1 to linear elements exhibit hierarchical dependency (Quiogue et al., 2023). We show that enlargement of small puncta labeled with ZW10^CZW-1^ is inhibited after depletion of Nuf2^him-10^, indicating that Nuf2 is required for efficient linear element assembly. In contrast, purified RZZ–Spindly proteins can assemble into filaments *in vitro* (Pereira et al., 2018). RZZ–Spindly exhibits structural similarity to vesicle coat proteins (Mosalaganti et al., 2017), which polymerize to organize donor membranes into vesicles. Therefore, RZZ–Spindly oligomerization might drive linear element formation as it does with fibrous corona expansion (Kops and Gassmann, 2020), which is facilitated *in vivo* by additional factors like Nuf2. Future studies will be required to understand the assembly of linear elements based on outer kinetochore protein interactions.

In addition to the intrinsic ability of kinetochore proteins to polymerize, microtubules also contribute to linear element formation. We observe that linear elements maintain a constant and dynamic colocalization with the cortical microtubule network. Furthermore, microtubule depletion inhibits linear element growth at the time of NEBD. Previously, linear element structures have been shown to expand after treatment with nocodazole (Pereira et al., 2018; Wu et al., 2023). We resolve this apparent discrepancy by showing that cells arrested in prometaphase I after APC/C inactivation can eventually form long linear elements even when microtubules are depleted. How microtubules affect the size of linear elements is unclear. The lack of linear element growth without Nuf2^him-10^ might reflect a requirement for plus-end microtubule binding in linear element assembly in addition to its role in assembling kinetochore structures. Collectively, our results suggest that the intrinsic ability of RZZ–Spindly to polymerize is enhanced by microtubule-dependent mechanisms *in vivo*. Multiple microtubule-interacting proteins are observed at linear elements that might facilitate linear element formation. RZZ–Spindly is well known for its role in recruiting dynein to kinetochores (Gama et al., 2017), which might also be involved in linear element assembly. Our finding that Nuf2^him-10^ depletion causes formation of small and motile linear element puncta might reflect residual dynein-mediated movements. Future studies will be important to understand how microtubules contribute to linear element formation.

As linear elements form just prior to NEBD and become undetectable during anaphase I, we suspect that they might be under the regulatory control of a CDK that is first activated in the cytoplasm (Jackman et al., 2003). We find that inhibition of PLK-1 significantly reduces linear element formation, which could reflect a direct role in linear element formation and/or be due to its role in CDK-1 regulation (Pintard and Archambault, 2018). Recently, CDK-1 and CKS-1 have also been found to localize to linear elements (Yang et al., 2026). The fibrous corona can be detached from chromosomes after microtubule and CDK-1 inhibition (Kops and Gassmann, 2020; Pereira et al., 2018; Sacristan et al., 2018). Previously, dynein has been observed on linear element-like structures, which appear extremely large when APC/C, microtubules and CDK are inhibited (Crowder et al., 2015). APC/C and CDK-1 regulate motor activity to control spindle positioning and rotation during meiosis I, are required for cytoplasmic streaming and affect cortical granule distribution (Bembenek et al., 2007; Ellefson and McNally, 2009, 2011; Yang et al., 2003). Whether APC/C and CDK-1 affect cytoplasmic organization through linear elements in addition to their roles at the spindle is an important question for future studies. The interplay between microtubule dynamics, cell signaling and kinetochore protein polymerization likely control linear element behavior and function.

An important question is to understand how linear elements couple microtubules to vesicle transport. Linear elements initially form in close contact with the ER and microtubules, and gradually associate with vesicles as they assemble into larger structures. The observation that ZW10 exists in an ER-tethering complex supports a model where linear elements first form at the ER (Arasaki et al., 2006; Hirose et al., 2004). As linear elements assemble, they appear to explore the cytoplasm, dynamically interacting with vesicles as they form clusters. This behavior might be similar to kinetochore behaviors during chromosome congression, when expanded fibrous corona material organizes microtubules to guide chromosomes to the spindle equator (Heald and Khodjakov, 2015; Magidson et al., 2015; Sikirzhytski et al., 2018). Given that different kinetochore proteins can enable microtubule binding at the plus- and minus-ends as well as laterally (Cheeseman and Desai, 2008; Wu et al., 2023), each of these modalities might contribute to vesicle transport. Vesicles could be transported by motors, such as dynein, which is well known for its role in vesicle transport (Reck-Peterson et al., 2018), that operate on the microtubule network, on linear elements or both. Future studies using precise mutations that can uncouple linear element formation from microtubule and motor function will be required to tease out these mechanisms. Studies of linear elements might reveal insights into vesicle transport mechanisms and serve as a useful context to further understand kinetochore protein function and behavior.

## MATERIALS AND METHODS

### Maintenance of transgenic worm strains

Multiple *C. elegans* strains were isolated and maintained using traditional methods as per Wormbook protocols (https://wormbook.org/). Unless otherwise noted, worms were maintained at 20°C and fed OP50 supplied by the *Caenorhabditis* Genetics Center (CGC). Some strains were obtained from the CGC. Transgenic lines generated for this project are listed in Table S1.

### RNAi treatments

Feeding RNAi was conducted using bacterial strains from the Ahringer library (Kamath and Ahringer, 2003). KNL-1 RNAi was provided by the Bowerman laboratory, University of Oregon (Quiogue et al. 2023). For feeding RNAi, L4 hermaphrodites were plated onto 60 mm agar NGM plates seeded with IPTG-induced RNAi bacterial strains at 20°C. Resultant phenotypes were assessed after 20–48 h of treatment.

### Auxin treatments

For auxin treatment, synchronized L4s from the strain EU3383 (Table S1) were fed OP50 on NGM plates containing 1 µM auxin (MP Biomedicals, 102037) for 13–16 h at 20°C.

### Staining procedures

#### Nile Blue staining

The permeability of embryos was assessed via protocol previously described (Carvalho et al., 2011). 150 µg/ml Nile Blue A stain was added to the medium prior to pouring. Plates were seeded with HT115 bacteria harboring the RNAi construct as described above. L4 worms were fed various RNAi strains over the course of 24 h and allowed to lay embryos. Animals were removed and placed onto fresh RNAi seeded plates containing Nile Blue A and allowed to lay embryos for another 24 h until removal. Laid embryos were left on the plate for 24 h after parent removal and were assessed for blue staining and embryo lethality using a SMZ745 Nikon Stereomicroscope under white light. Embryos containing intact eggshells were impermeable to the dye, and embryos with defective eggshells were stained blue. Proportions of stained embryos, unstained embryos and hatched larvae were calculated as a percentage of the total brood for each experiment.

#### FM2-10 staining

N2 worms at L4 stage were treated with RNAi via feeding for 40–46 h, at 20°C or 25°C. For auxin treatment, the strain EU3383 (Table S1) was fed OP50 on NGM plates containing 1 µM auxin for 13–16 h. Following treatment, embryos were dissected into a 15 µl droplet of 9 µM FM2-10 (Invitrogen) diluted into Schneider's medium on a clean glass slide. The mount was placed between a 1.75 mm silicone tissue imaging chamber and sealed with a glass coverslip. Imaging with an inverted confocal microscope immediately followed.

### Live-cell imaging

Live-cell imaging data of JAB19, JAB274, JAB276, JAB280, JAB285, JAB286, DKC547 were collected on an inverted Nikon Eclipse Ti2-E with a 60×1.42NA objective and 100×/NA 1.45 objective, a CSU-X1 spinning disk system, and Andor iXon Life camera operated by NIS-Elements software (Nikon). Unless otherwise mentioned, live-cell imaging was conducted at room temperature.

Live imaging of KLP-19 (FGP36), CDC-20 (OD2591), and KNL-3 (FGP722) was done using a CFI Plan Apochromat Lambda 60×/NA 1.4 oil objective mounted on a microscope (Nikon Eclipse Ti) equipped with a Prime 95B 22 mm camera (Photometrics), a spinning-disk head (CSU-X1; Yokogawa Electric Corporation).

Acquisition parameters were controlled with NIS software (Nikon).

Live imaging of HCP-4 (FGP311), NDC-80 (FGP372), KNL-1 (FGP517), MDF-1 (OD2920), MDF-2 (OD216), BUB-1 (FGP202), BUB-3 (TG4193), KLP-7 (FGP261), CLS-2 (JDU38) was done using a 60×/NA 1.4 oil objective mounted on a microscope (IX81; Olympus), an EMCCD Cascade II camera (Photometrics), spinning-disk head (CSU-X1; Yokogawa Electric Corporation). Acquisition parameters were controlled by MetaMorph seven software (Molecular Devices).

For all live imaging experiments, full or partial projections and single planes are presented. Files were stored, classified and managed using OMERO (Allan et al., 2012). Figures were prepared using OMERO.figure and assembled using Adobe Illustrator. Image analysis and manipulation was performed in Fiji software (National Institutes of Health).

#### *In utero* live-cell imaging

A chemical immobilization method was applied by mounting worms in an M9 solution containing 5mM levamisole (2% solution; Tokyo Chemical Industry, T1215) on a 2% agarose pad affixed to a glass slide and sealed with Vaseline, following standard protocol (Bembenek et al., 2007).

#### *Ex utero* time-lapse imaging of meiosis I

Oocytes were dissected and mounted in 5 µl of L-15 blastomere culture medium [0.5 mg/ml inulin (Sigma-Aldrich, I2255); 25 mM HEPES, pH 7.5 (Sigma-Aldrich, H3375) in 60% Leibowitz L-15 medium (Thermo Fisher Scientific, 11415049) and 20% heat-inactivated FBS] on 24×40 mm #1.5 coverslips. Once dissection was performed and early oocytes were identified using a stereomicroscope, a circle of Vaseline was laid around the sample, and a custom-made plastic holder (with a centered window) was placed on top of the coverslip. The sample was imaged immediately.

### Quantifications

#### Fluorescence

Fluorescent values for the linear element and chromosome versus cytoplasm curve in Fig. 1 were acquired from single-plane *in utero* movies of maturing −1 oocytes using the same acquisition settings. Three measurements for chromosomes, linear elements, cytoplasm and background were taken at each timepoint, using the mean value of a 3×3 pixel diameter selection area (for chromosomes and linear elements) or a 15×15 pixel diameter selection area (for cytoplasm and background). The mean background intensity was calculated and subtracted from each measurement of linear elements, chromosomes and cytoplasm. The background-subtracted measurements were then averaged and expressed as ratios between each other.

#### Linear element length

The timepoint for the beginning of the puncta, intermediate, long and condensed stage were recorded for every timeseries of ovulation for the CZW-1::GFP, CPG-2::mCherry strain grown on OP50. Time-lapse movies were acquired of linear element formation with CZW-1::GFP. We defined four stages of linear element formation. The puncta stage starts when a burst of numerous puncta form and ends when most linear elements in view appeared longer than puncta. The intermediate stage lasts until the first sign of long linear elements appear, as cross sections of linear elements in a single plane can be confused when most of them become long. The condensed stage was also marked by the first observation of a condensed linear element. Ideal timepoints were selected from these stages with as many of the linear elements in focus as possible. Using the segmented line tool in ImageJ, we measured the length along the longest distance across each linear element in focus. For branched linear elements, the total length included the sum of all branches (which is less than 10 examples out of the total measured for the long stage). For mutant comparisons in Fig. 5J, the linear elements were measured at the timepoint when they appeared longest for each movie. For the ‘condensed’ stage, some data points were excluded to avoid artifacts caused by extensive sheath cell contractions which occur prior to ovulation that distort the images.

### Distance analysis

Raw imaging files were quantified in ImageJ using the Distance Analysis (DiAna) plugin (https://imagej.net/plugins/distance-analysis). Extraneous background signal was cropped from files prior to initializing the threshold mask for assigning boundaries to cellular objects. Live-imaging files of singular oocytes from linear element formation through to ovulation of the gravid CZW-1::GFP, CPG-2::mCherry strain were used in this analysis. Movies where either of the two labels were extensively photobleached were excluded. Files were split into separate color channels, and an edge-to-edge analysis was performed on all objects within each channel containing signal above the threshold. Distances between each linear element (designated as the primary object A) and the closest edge-to-edge distance to a vesicle (object B) were measured.

#### Statistics

For linear element length and distance to the nearest vesicle measurements in Figs 1, 2 and 5, the median of all measurements was recorded for each individual oocyte. If multiple ovulations from the same worm were observed, only the first oocyte was used for analysis across the datasets. The medians from 5–30 oocytes (depending on the dataset) were then compared using one way ANOVA, followed by a post hoc Tukey HSD test (Figs 1F and 2H) or Dunnett's test to compare to control (Fig. 5J,O).

For the Nile Blue data, brood size was calculated as the sum of the embryos and larvae present on a 35 mm NGM plate 48 h after feeding. For each RNAi condition, the fraction of white/blue embryos per experiment were averaged across 2–10 experiments and plotted as a stacked column. Error bars display the standard error of the mean (s.e.m.) for white and blue embryos for each condition. Statistical significance was calculated using one-way ANOVA followed by a post hoc Dunnett's test.

For FM2-10 data, a hanging drop mount was constructed by affixing a chemically inert silicone tissue imaging chamber (Thermo Fisher Scientific #V11333) between a glass coverslip and glass slide. Three to five gravid adults were dissected into a 15 µl droplet of Schneider's medium containing 9 mM FM2-10 and were placed onto the microscope stage immediately after for embryo counting. The number of permeable embryos with internal membrane staining were plotted as a fraction of the total and displayed as a bar chart. Embryos in meiosis II or earlier at the time of dissection were removed from this calculation due to their innate permeability at this stage. The percentage of stained embryos from each experiment was compared using one-way ANOVA followed by a post hoc Dunnett's test.

Across all charts, statistically significant conditions are marked with asterisks (*P<0.05, **P<0.01, ***P<0.001). Statistical analysis was performed using Microsoft Excel or JASP (https://jasp-stats.org/).

## Supplementary Material



10.1242/joces.264478_sup1 Supplementary information
