# Correction: Outer kinetochore proteins form linear elements to regulate vesicle transport

**DOI:** 10.1242/jcs.265311

**Published:** 2026-10-16

**Authors:** Shane J. Kowaleski, Alexis Bridgewater, Cody Saraceno, Miranda Dudek, Federico Pelisch, Joshua N. Bembenek

There were errors in *J. Cell Sci.* (2026) **139**, jcs264478 (doi:10.1242/jcs.264478).

An Introduction paragraph was missing some relevant references and an example from *Xenopus*. The original and corrected versions are shown below.

**Corrected:**

Separase localizes to kinetochores and linear elements at early stages of division, and only moves to sites of action at the midbivalent and vesicles at anaphase onset (Bembenek et al., 2007; Sorensen Turpin et al., 2025). Numerous outer kinetochore proteins have also been shown to localize to cytoplasmic linear element structures in *C. elegans* (Bellutti et al., 2024; Bembenek et al., 2007; Danlasky et al., 2020; Dumont et al., 2010; Hattersley et al., 2016; Hattersley et al., 2022; Howe et al., 2001; Macaisne et al., 2023; Monen et al., 2005; Pereira et al., 2018; Pitayu-Nugroho et al., 2023; Quiogue et al., 2023; Taylor et al., 2023), *Drosophila* (Gilliland et al., 2007, 2024, 2009; Shapiro et al., 2025) and bovine oocytes (Wu et al., 2023). Expanded linear kinetochore structures have also been observed in *Xenopus* egg extracts (Wynne and Funabiki, 2015).

**Original:**

Separase localizes to kinetochores and linear elements at early stages of division, and only moves to sites of action at the midbivalent and vesicles at anaphase onset (Bembenek et al., 2007; Sorensen Turpin et al., 2025). Numerous outer kinetochore proteins have also been shown to localize to cytoplasmic linear element structures in *C. elegans* (Dumont et al., 2010; Hattersley et al., 2016, 2022; Howe et al., 2001; Monen et al., 2005; Pelisch et al., 2017; Pereira et al., 2018; Quiogue et al., 2023; Schlientz and Bowerman, 2020; Taylor et al., 2023), *Drosophila* (Gilliland et al., 2007, 2024, 2009) and bovine oocytes (Wu et al., 2023).

A Results paragraph on page 2 was missing some relevant references. The original and corrected versions are shown below.

**Corrected:**

Numerous outer kinetochore components and related cell cycle regulators have been reported to associate with linear elements, as summarized in Table S2 (Bai and Bembenek, 2017; Bellutti et al., 2024; Bembenek et al., 2007; Crowder et al., 2015; Danlasky et al., 2020; Divekar et al., 2021; Dumont et al., 2010; Gigant et al., 2017; Hattersley et al., 2016; Hattersley et al., 2022; Howe et al., 2001; Laband et al., 2017; Macaisne et al., 2023; Monen et al., 2005; Mullen and Wignall, 2017; Muscat et al., 2015; Pelisch et al., 2017; Pereira et al., 2018; Pitayu-Nugroho et al., 2023; Quiogue et al., 2023; Schlientz and Bowerman, 2020; Sorensen Turpin et al., 2025; Taylor et al., 2023; Wang et al., 2013; Yang et al., 2026).

**Original:**

Numerous outer kinetochore components and related cell cycle regulators have been reported to associate with linear elements, as summarized in Table S2 (Bai and Bembenek, 2017; Bellutti et al., 2024; Bembenek et al., 2007; Crowder et al., 2015; Dumont et al., 2010; Gigant et al., 2017; Hattersley et al., 2016, 2022; Howe et al., 2001; Monen et al., 2005; Pelisch et al., 2017; Pereira et al., 2018; Quiogue et al., 2023; Schlientz and Bowerman, 2020; Sorensen Turpin et al., 2025; Taylor et al., 2023; Wang et al., 2013; Yang et al., 2026).

In two instances in the Results, the *N*-value was corrected to report the number of times the phenotype was observed and the total sample size, as follows.

The text ‘To gain insight into what stimulates their formation at NEBD, we RNAi-depleted the cell cycle regulator and linear element component PLK-1 and found linear element formation was significantly inhibited (*N*=11 or 12; Fig. S2).’ has been corrected to give the *N*-value as ‘*N*=11/12 embryos examined’.

The text ‘Crucially, vesicles remained trapped in the cytoplasm after the majority underwent exocytosis in anaphase I (*N*=10 or 11, Fig. S3K).’ has been corrected to give the *N*-value as ‘*N*=10/11 embryos examined’.

In the Materials and Methods, the concentrations used for two reagents were given incorrectly.

In the ‘Auxin treatments’ and ‘FM2-10 staining’ sections, NGM plates were described as containing 1 μM auxin, which has been corrected to read 1 mM.

In the ‘Nile Blue staining’ section, it was stated Nile Blue A stain was used at 150 μg/ml, which has been corrected to read 430 μg/ml.

In addition, the ‘FM2-10 staining’ section now clarifies the dilution into Schneider's medium as 1:10, which was missing from the original version, to give corrected text of ‘Following treatment, embryos were dissected into a 15 μl droplet of 9 μM FM2-10 (Invitrogen) diluted 1:10 into Schneider's medium on a clean glass slide.’

In Fig. 4G, the final timestamp was corrected to ‘6.3’.

In Fig. 6J, the inset overlay box position was corrected.

In Table S2, new citations were added for multiple rows.

Original and corrected versions of these items are shown below.

**Fig. 4G (corrected panel).**
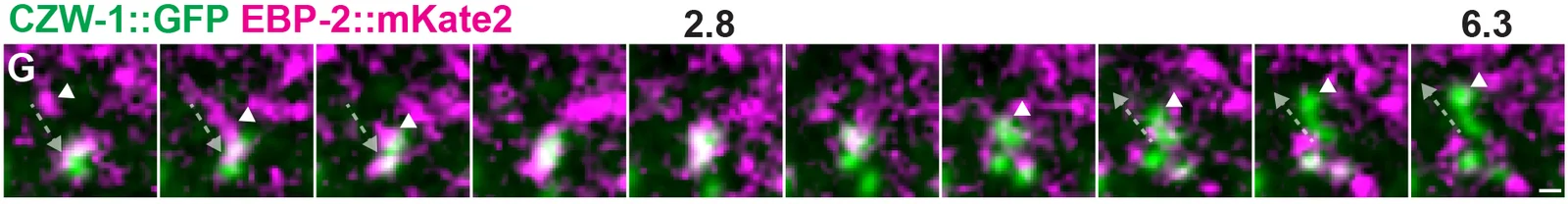
(G) Montage of EBP-2::mKate2 (+ end microtubule marker, magenta) and CZW-1::GFP (green) during linear element assembly. Arrowheads indicate a, EBP-2::mKate2 spot (magenta) moving toward the linear element (left, dashed arrow shows trajectory), and slightly later the linear element (right, arrowheads) moves the reverse direction (dashed arrow). Time interval is 700 ms. Scale bar: 1 µm.

**Fig. 4G (original panel).**
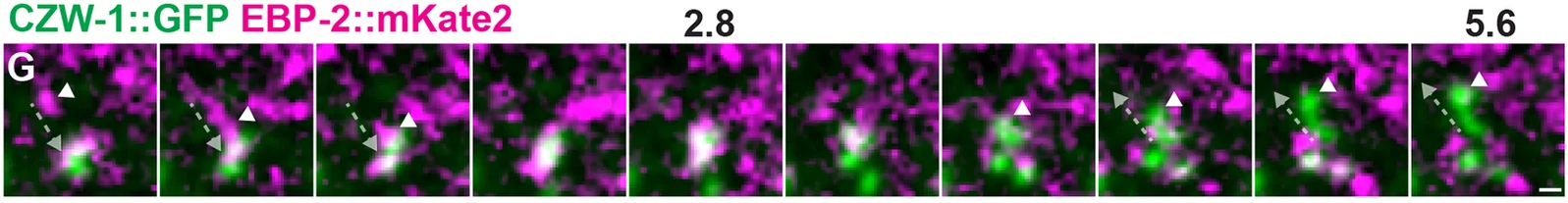
(G) Montage of EBP-2::mKate2 (+ end microtubule marker, magenta) and CZW-1::GFP (green) during linear element assembly. Arrowheads indicate a, EBP-2::mKate2 spot (magenta) moving toward the linear element (left, dashed arrow shows trajectory), and slightly later the linear element (right, arrowheads) moves the reverse direction (dashed arrow). Time interval is 700 ms. Scale bar: 1 µm.

**Fig. 6J (corrected panel).**
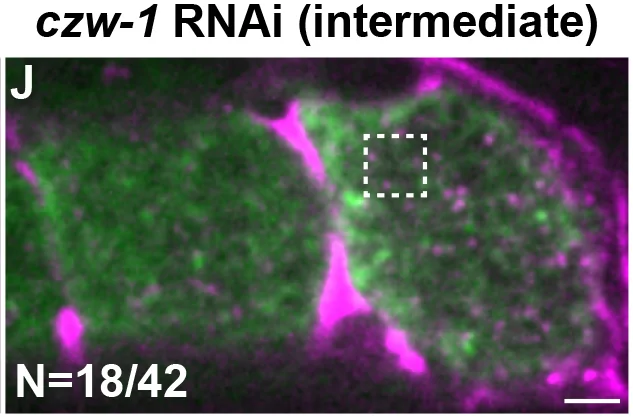
(I–K) Representative images of ER (SP12::GFP, green) and cortical granule (CPG-2::mCherry, magenta) distribution in the germline of worms fed control (empty vector) RNAi (I) or czw-1(RNAi) (J,K). After 24–25 h of feeding, we observed an intermediate czw-1(RNAi) phenotype in 18/42 cases where CPG-2::mCherry appears in smaller puncta (J), whereas in severe cases (K, 11/42 animals imaged), most CPG-2::mCherry signal remains trapped in the ER. Scale bars: 5 µm (main images), 1 µm (magnifications).

**Fig. 6J (original panel).**
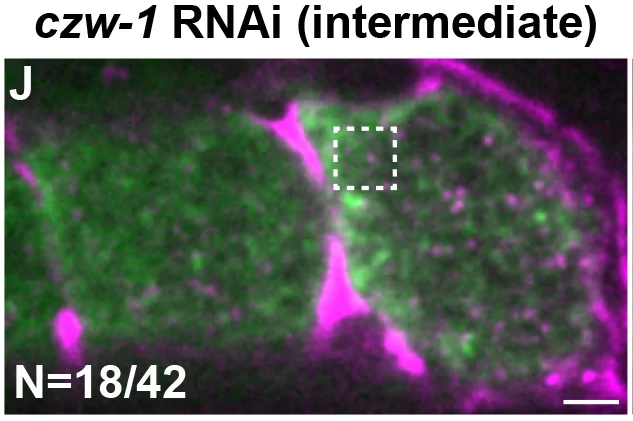
(I–K) Representative images of ER (SP12::GFP, green) and cortical granule (CPG-2::mCherry, magenta) distribution in the germline of worms fed control (empty vector) RNAi (I) or czw-1(RNAi) (J,K). After 24–25 h of feeding, we observed an intermediate czw-1(RNAi) phenotype in 18/42 cases where CPG-2::mCherry appears in smaller puncta (J), whereas in severe cases (K, 11/42 animals imaged), most CPG-2::mCherry signal remains trapped in the ER. Scale bars: 5 µm (main images), 1 µm (magnifications).

**Figure:**
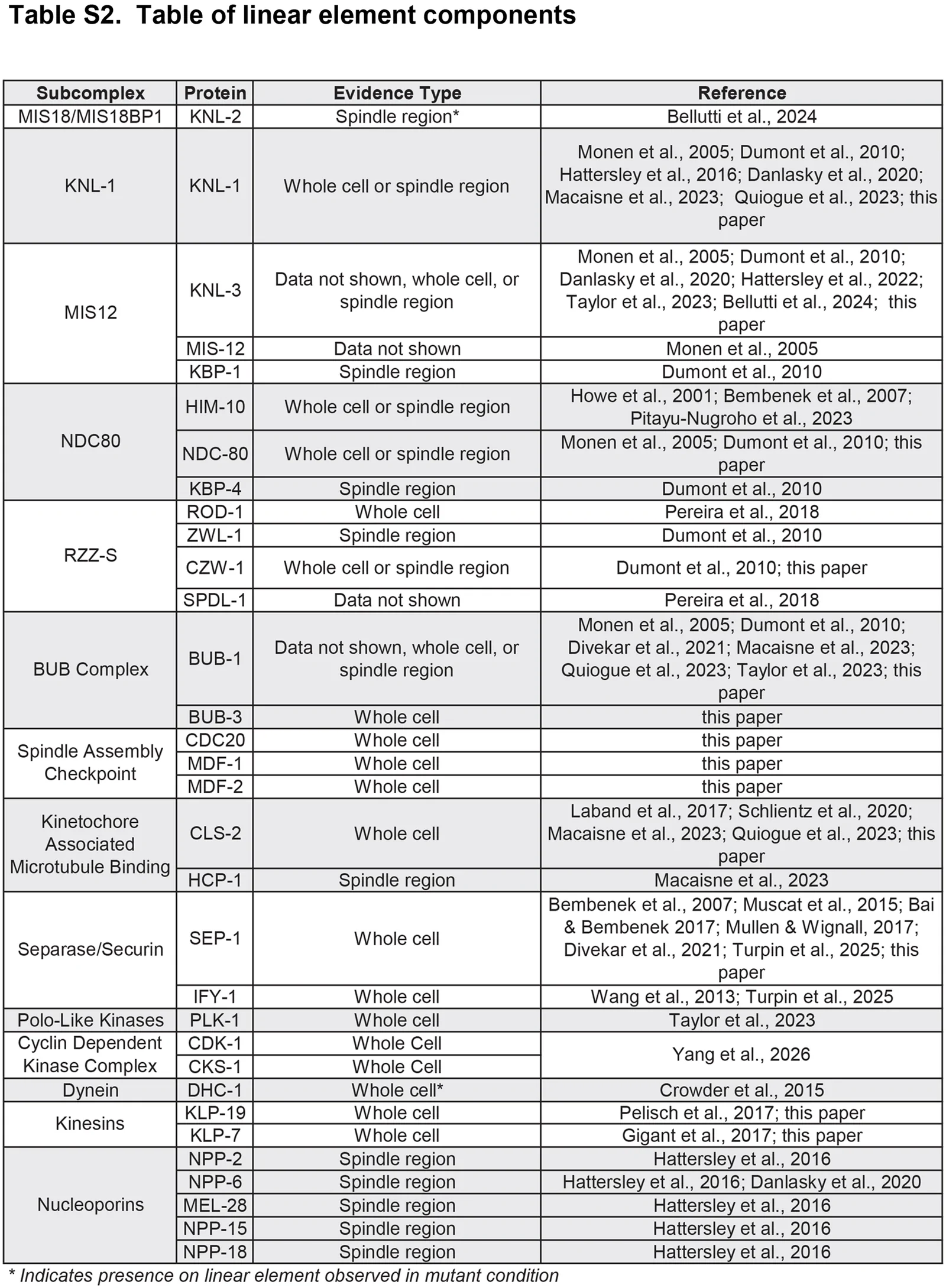


**Figure:**
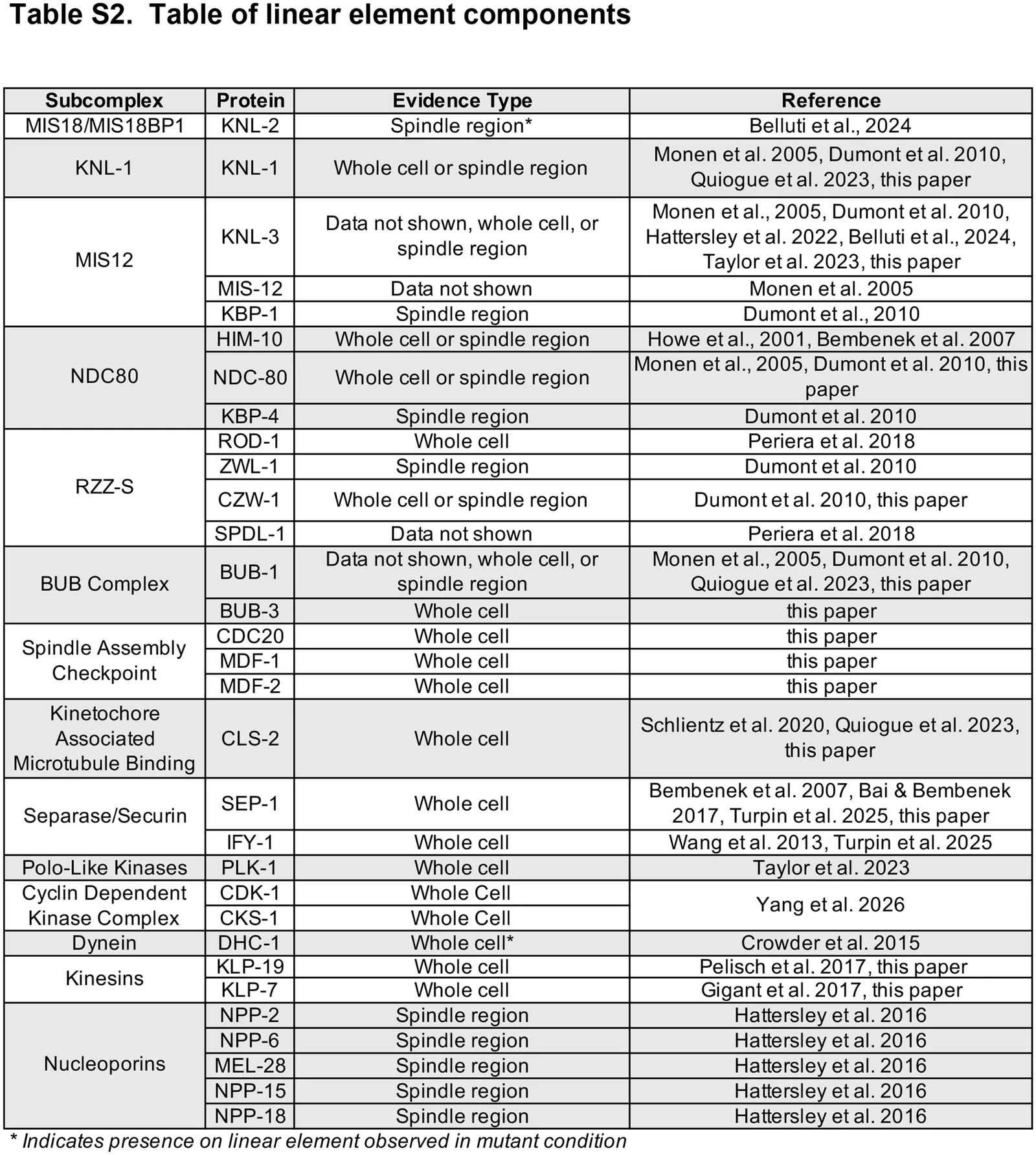


**Table S2 (corrected).**

**Table S2 (orginal).**

## Notes

These errors have been corrected in the online and pdf versions of the paper. The authors apologise for any inconvenience caused.
