# Supplementary material for: Outer kinetochore proteins form linear elements to regulate vesicle transport
Source: J Cell Sci. 2026 May 11;139(9):jcs264478. doi: 10.1242/jcs.264478 (PMC13245919; doi:10.1242/jcs.264478)
Supplement: Supplementary information [file joces-139-264478-s1.pdf]

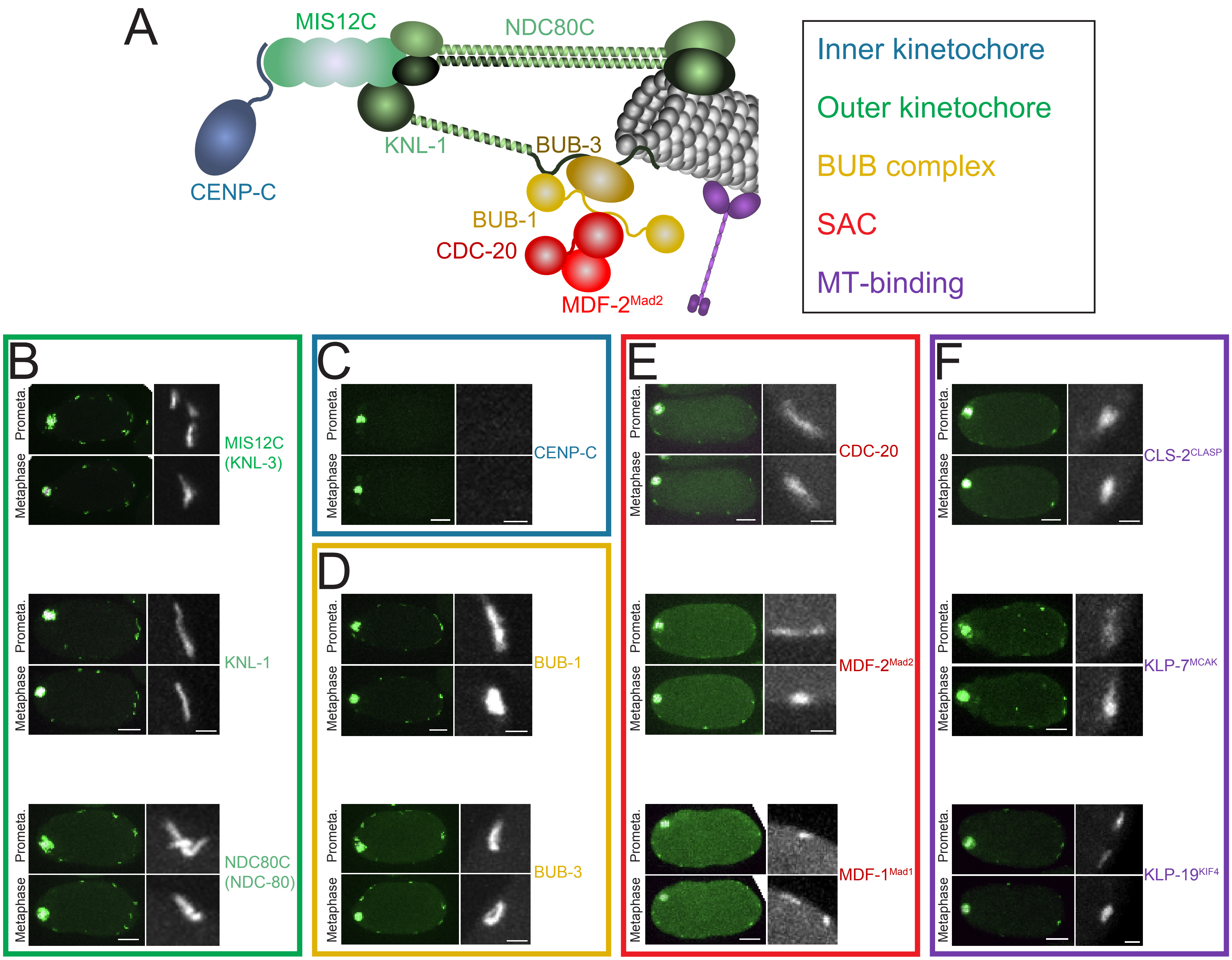

Fig. S1. Analysis of Linear Elements Composition.

(A) Schematic of the different proteins or protein complexes analyzed. (B-F) Prometaphase and Metaphase oocytes are shown for each component. A zoomed image of a single linear element is shown on the right of each oocyte. Scale bars, 10 µm (full oocytes) and 2 µm (zoomed images). (B) Components of the KMN network. (C) Inner kinetochore. (D) The BUB complex. (E) SAC components that form the mitotic checkpoint complex (MCC). (F) Microtubule-binding proteins.

Journal of Cell Science • Supplementary information

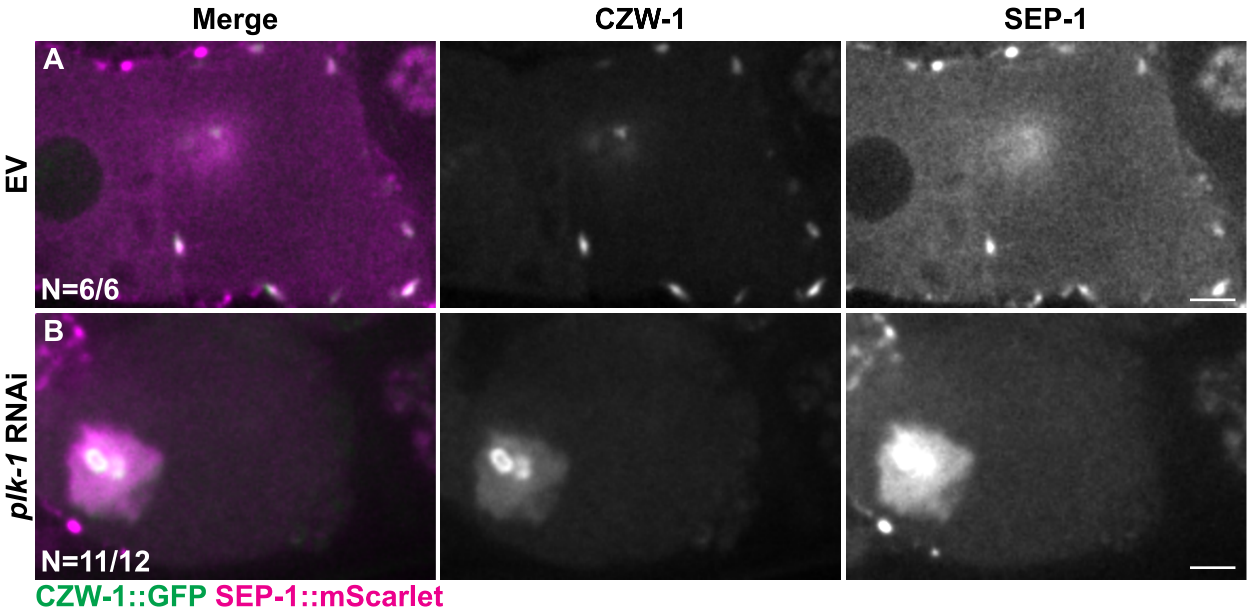

Fig. S2. Linear Element Formation is Inhibited under *plk-1*.

(A-B) Just prior to ovulation, linear elements are present in empty vector control (A, N=6/6) and contain both SEP-1::mScarlet and CZW-1::GFP but are absent in most oocytes treated with *plk-1(RNAi)* through ovulation (B, N=11/12). Scale Bars: 5 µm.

Journal of Cell Science • Supplementary information

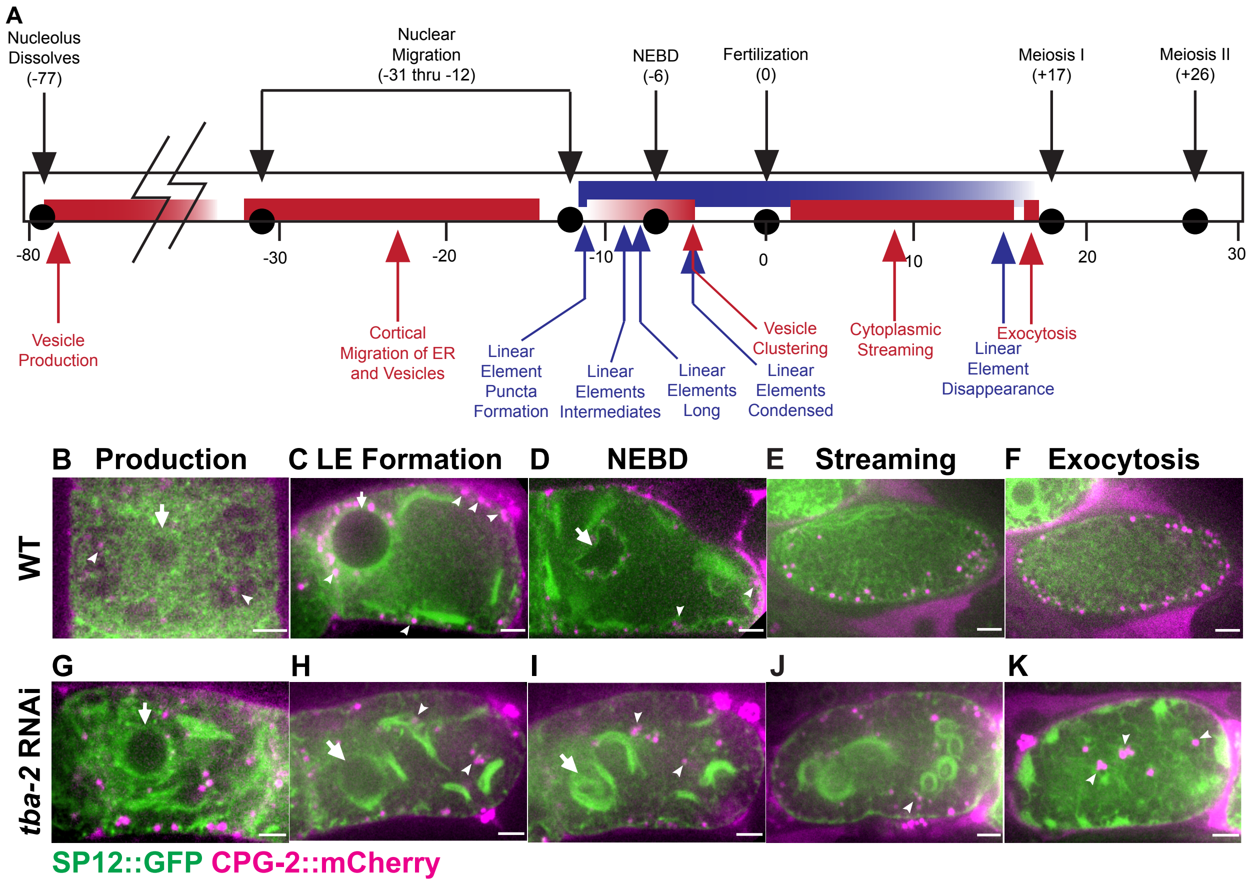

Fig. S3. Global dynamics of ER and Cortical Granules during Meiosis I

(A) Timeline of events during oocyte meiosis. Upper events are based on previous studies (McCarter et al., 1999), events below are based on our observations of ER and vesicles during these different time periods. Red highlights vesicle events, blue indicates linear element timeline. (B-F) Representative images showing ER morphology and vesicle distribution at different stages of oocyte meiosis I. Arrows indicate position of the nucleus, which moves posteriorly before linear element formation. Arrowheads indicate cortical granules. (G-K) Organization of the ER and vesicles in the cytoplasm is significantly disrupted in *tba-2(RNAi)* oocytes at each stage of meiosis I. Arrows indicate nucleus, arrowheads indicate vesicles that are not cortically displaced in (H-J) and remain in the cytoplasm after they should normally undergo exocytosis in (K).

Journal of Cell Science • Supplementary information

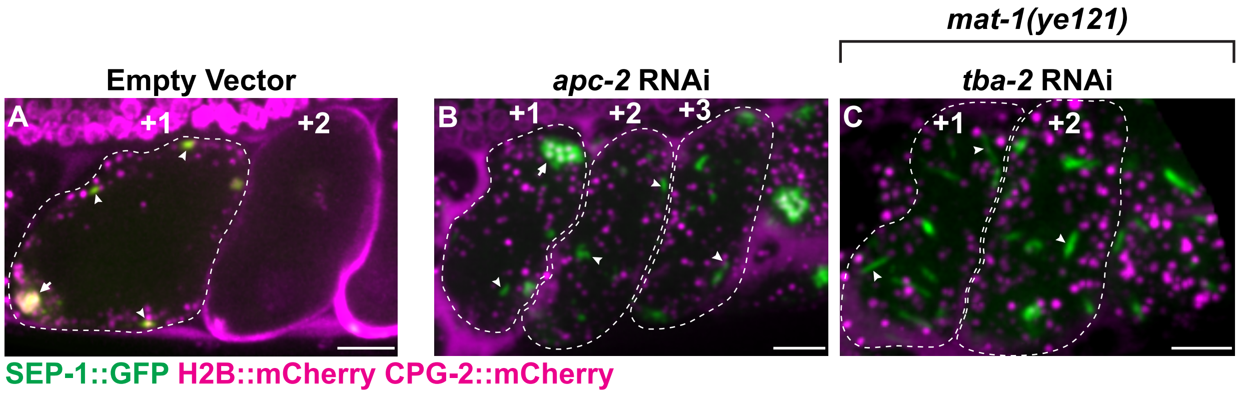

Fig. S4. Linear Elements in APC/C Arrested Embryos

(A-C) Maximum Z projections of embryos expressing SEP-1::GFP to show linear elements, H2B::mCherry to label chromosomes, and CPG-2::mCherry in cortical granules. Scale bars are 5 µm. (A) Control embryos have numerous cortical granules throughout the cortex with multiple linear elements (arrowheads) during metaphase I ($^{+}$1 embryo, indicated by dashed outline). +2 and older embryos show CPG-2::mCherry incorporated into the eggshell after exocytosis and linear elements are not observed. (B) *apc-2(RNAi)* embryos arrested in prometaphase I retain cortical granules in the cytoplasm and numerous linear elements (arrowheads) are observed. (C) *mat-1(ye121); tba-2(RNAi)* embryos arrested in prometaphase I also do not secrete CPG-2::mCherry. Long term arrested embryos (+1, +2, etc.) have numerous linear elements (arrowheads) that appear longer than normal.

Journal of Cell Science • Supplementary information

Table S1. List of *C. elegans* strains used in this study.

Strain	Genotype
JAB19	*ojIs23,unc-119(ed3);ojIs23[sp12::GFP unc-119(+)];Itls151[p5033;Pcpg-2::cpg1SigSeq::mCherry-TEV-STag::cpg-2;unc119(+)]*
JAB274	*cav-1(erb-78[cav-1::GFP]); ItIs151 [pSO33; Pcpg-2::cpg-1SigSeq::mCherry-TEV-STag::cpg-2; unc- 119(+)]*
JAB276	*unc-119(ed3)III;Itls151[p5033; Pcpg-2::cpg-1SigSeq::mCherry-TEV-STag::cpg-2;unc-119(+)];czw-1::GFP*
JAB280	*ruls57[pie-1p::GFP::tubulin+unc-119(+)];knl-1(dha110(TagRFP::knl-1))III*
JAB285	*sep-1(erb-84[sep-1::linker::mScarlet]);czw-1::GFP*
JAB286	*ojls23, unc-119(ed3);ojls23[sp12::GFP unc-119(+)];knl-1(dha110(TagRFP::knl-1))III*
DKC547	*knl-1(dha110(TagRFP::knl-1))III*
FGP311	*hcp-4(lt72[GFP::hcp-4])I; ltIs37 [pAA64; pie1/mCHERRY::his-58; unc-119 (+)]IV)I; ieSi65 [sun-1p::TIR1::sun-1 3'UTR + Cbr-unc-119(+)] II; unc119(ed3)III*
FGP372	*ndc-80(lt54[ndc-80::GFP::tev::loxP::3xFlag])IV; ltIs37 [pAA64; pie-1/mCHERRY::his-58; unc-119 (+)]IV*
FGP517	*knl-1(lt53[knl-1::GFP::tev::loxP::3xFlag]))III; ItIs37 [Ppie-1::mCherry::his-58 (pAA64); unc-119(+)] iv; unc-119(ed3)???*

Journal of Cell Science • Supplementary information

FGP722	*knl-3(dha19 [mscarlet-I^3XFLAG::knl-3] V; ruIs32 [pie-1::GFP::histone + unc-119(+)], unc-119 (ed3)*
OD2591	*ltSi814[pPLG047; Pfzy-1::gfp::fzy-1::fzy-1 3'UTR; cb-unc-119(+)]I; unc-119(ed3)III?;; ltIs37 [pAA64; pie-1/mCHERRY::his-58; unc-119 (+)] IV*
OD2920	*unc-119(ed3)?III; ltIs37[pAA64; pie-1/mCherry::his-58; unc-119 (+)]IV; mdf- 1(lt39[gfp::tev::loxP::3xFlag::mdf-1])V*
OD216	*unc-119(ed3) III; ltIs52 [pOD379; pie-1/GFP::Y69A2AR.30; unc-119 (+)], ltIs37 [pAA64; pie-1/mCherry::his-58; unc-119 (+)] IV, mdf2(tm2190)*
FGP202	*bub-1(syb1134[bub-1::linker::gfp])I; ltIs37 [pAA64; pie-1/mCherry::his-58; unc-119 (+)]IV; unc119(ed3)III*
TG4193	*bub-3(knu207[Pbub-3::eGFP::bub-3::3?UTRbub-3)]; odIs57[Ppie-1::mCherry::histonehistone + unc-119(+)]; unc-119(ed3)*
FGP261	*klp-7(cp178[klp-7::mNG-C1^3xFlag])III; ltIs37 [pAA64; pie-1/mCHERRY::his-58; unc-119 (+)]IV*
FGP36	*klp-19(fgp2[klp-19::loxP::GFP::FLAG::degron])III; ltIs37 [pAA64; pie-1p::mCherry::his-58 + unc-119(+)]*
JDU38	*unc-119(ed3) III; ijmSi3 [pJD342/pJD330; ChrI_5'mex-5_cls-2reenc::GFP_tbb-2; cb-unc-119(+)]I; unc-119(ed3)III?; ltIs37 [pAA64; pie1/mCHERRY::his-58; unc-119 (+)]IV.*
EU3383	*knl-1[or1982(degron::knl-1]III; ieSi38 [sun-1p::TIR1::mRuby::sun-1 3'UTR + Cbr-unc-119(+)] IV.*
JAB277	*[pie-1p:mCherry::sp12::pie-1 3'UTR + unc-119(+)]; jzIs49[pRK226(Ppie-1::GFP::czw-1); unc-119(+)]*
JAB299	*ebp-2(or1954[ebp-2::mKate2]) II; unc-119(ed3) (III); jzIs49[pRK226(Ppie-1::GFP::czw-1); unc-119(+)]*

Journal of Cell Science • Supplementary information

Table S2. Table of linear element components

Subcomplex	Protein	Evidence Type	Reference
MIS18/MIS18BP1	KNL-2	Spindle region*	Belluti et al., 2024
KNL-1	KNL-1	Whole cell or spindle region	Monen et al. 2005, Dumont et al. 2010, Quiogue et al. 2023, this paper
MIS12	KNL-3	Data not shown, whole cell, or spindle region	Monen et al., 2005, Dumont et al. 2010, Hattersley et al. 2022, Belluti et al., 2024, Taylor et al. 2023, this paper
	MIS-12	Data not shown	Monen et al. 2005
	KBP-1	Spindle region	Dumont et al., 2010
NDC80	HIM-10	Whole cell or spindle region	Howe et al., 2001, Bembenek et al. 2007
	NDC-80	Whole cell or spindle region	Monen et al., 2005, Dumont et al. 2010, this paper
	KBP-4	Spindle region	Dumont et al. 2010
RZZ-S	ROD-1	Whole cell	Periera et al. 2018
	ZWL-1	Spindle region	Dumont et al. 2010
	CZW-1	Whole cell or spindle region	Dumont et al. 2010, this paper
	SPDL-1	Data not shown	Periera et al. 2018
BUB Complex	BUB-1	Data not shown, whole cell, or spindle region	Monen et al., 2005, Dumont et al. 2010, Quiogue et al. 2023, this paper
	BUB-3	Whole cell	this paper
Spindle Assembly Checkpoint	CDC20	Whole cell	this paper
	MDF-1	Whole cell	this paper
	MDF-2	Whole cell	this paper
Kinetochore Associated Microtubule Binding	CLS-2	Whole cell	Schlientz et al. 2020, Quiogue et al. 2023, this paper
Separase/Securin	SEP-1	Whole cell	Bembenek et al. 2007, Bai & Bembenek 2017, Turpin et al. 2025, this paper
	IFY-1	Whole cell	Wang et al. 2013, Turpin et al. 2025
Polo-Like Kinases	PLK-1	Whole cell	Taylor et al. 2023
Cyclin Dependent Kinase Complex	CDK-1	Whole Cell	Yang et al. 2026
	CKS-1	Whole Cell	
Dynein	DHC-1	Whole cell*	Crowder et al. 2015
Kinesins	KLP-19	Whole cell	Pelisch et al. 2017, this paper
	KLP-7	Whole cell	Gigant et al. 2017, this paper
Nucleoporins	NPP-2	Spindle region	Hattersley et al. 2016
	NPP-6	Spindle region	Hattersley et al. 2016
	MEL-28	Spindle region	Hattersley et al. 2016
	NPP-15	Spindle region	Hattersley et al. 2016
	NPP-18	Spindle region	Hattersley et al. 2016

** Indicates presence on linear element observed in mutant condition*

Journal of Cell Science • Supplementary information

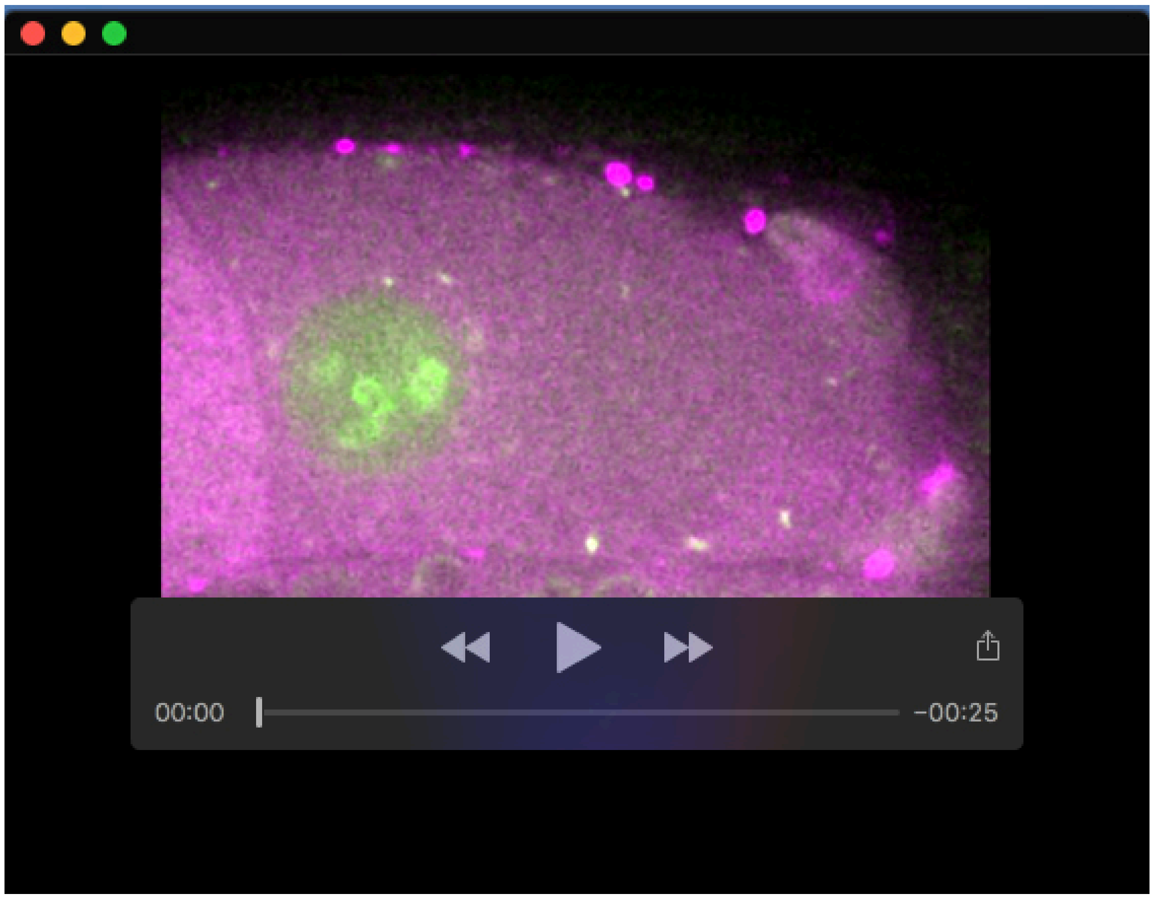

Movie 1. Linear Elements Assemble Just Before NEBD

CZW-1::GFP puncta (green) appear prior to NEBD and before SEP-1::mScarlet (magenta) accumulates on linear elements. Single plane images were captured every 5 seconds. Playback speed is 10 frames per second.

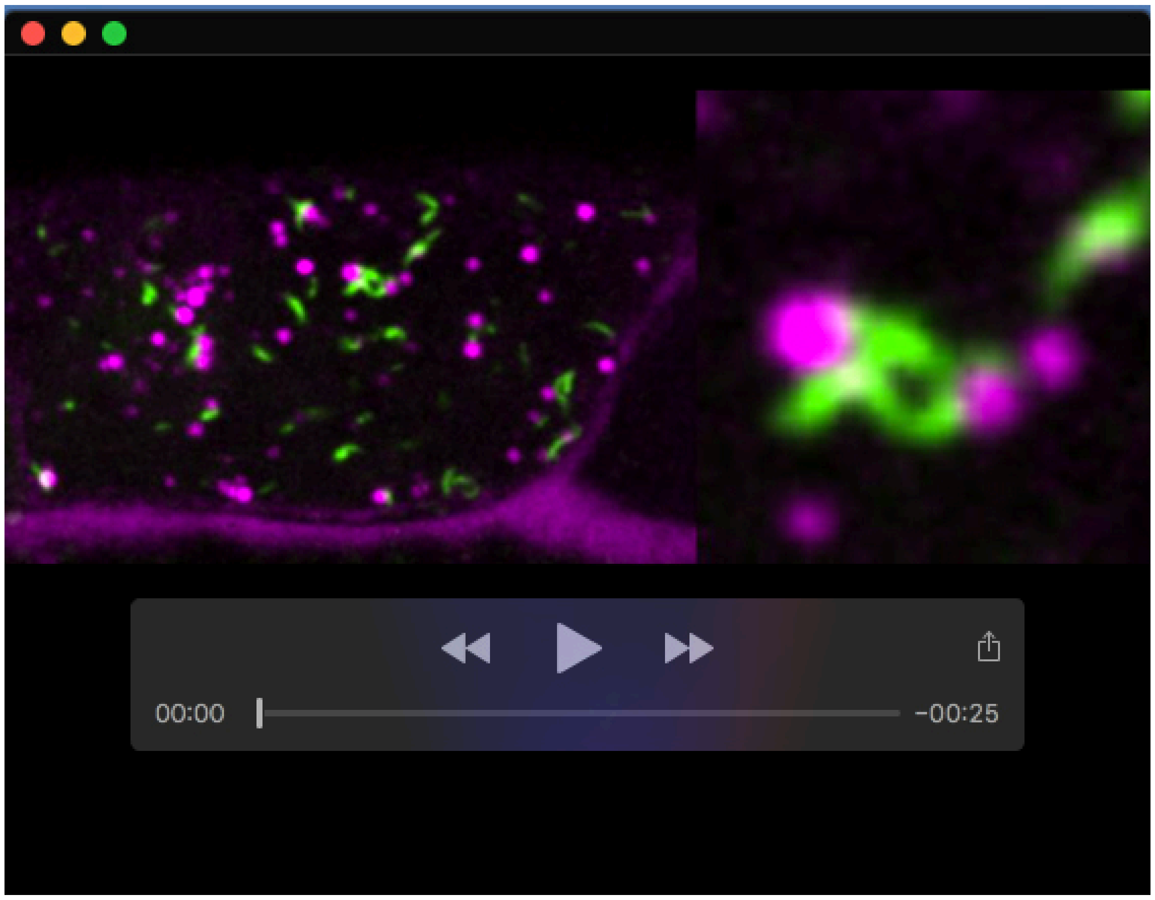

Movie 2. Linear Elements Associate with Cortical Granule Clusters

As linear elements (CZW-1::GFP, green) assemble, they form clusters with cortical granules (CPG-2::mCherry, magenta) at NEBD. Movie and inset are maximum intensity projections acquired every 5 seconds. Playback speed is 10 frames per second.

Journal of Cell Science • Supplementary information

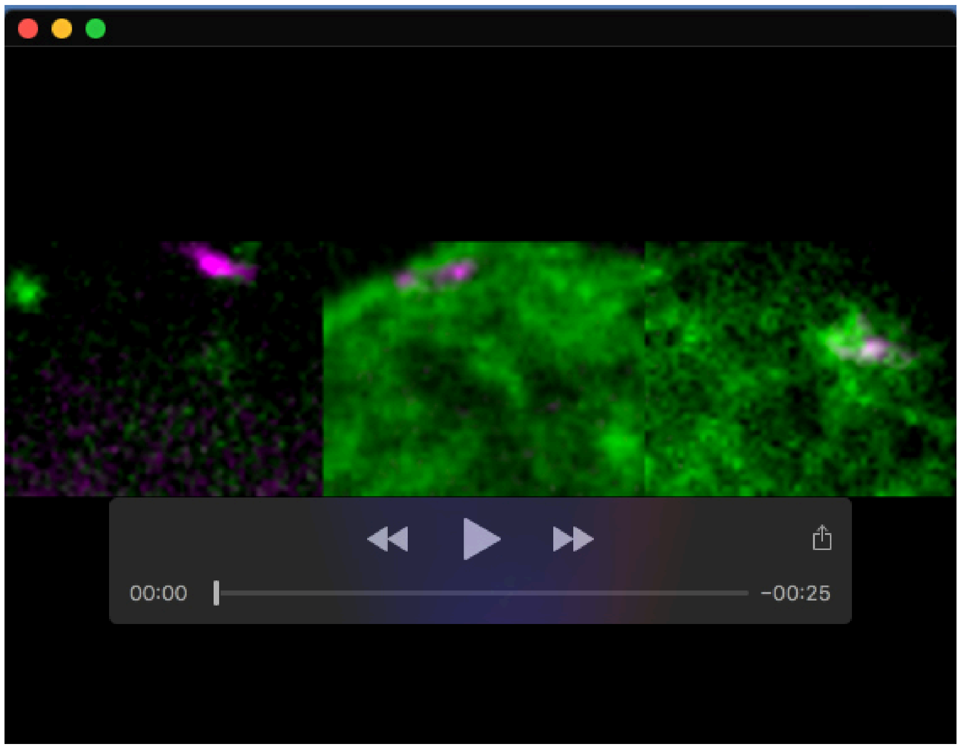

Movie 3. Linear Elements during Cytoplasmic Streaming

Linear elements (CZW-1::GFP or TagRFP::KNL-1, magenta) appear relatively static while cortical granules (CPG-2::mCherry, green, left panel), ER (SP12::GFP, green, middle panel) and microtubules (GFP::tubulin, green, right) move rapidly during cytoplasmic streaming in prometaphase I. Single plane images were acquired every 5 seconds for the CZW-1::GFP + CPG-2::mCherry (left) and TagRFP::KNL-1 + SP12::GFP (middle) movies, and every 10 seconds for the TagRFP::KNL-1 + GFP::tubulin (right) movie. Playback speed is 7 frames per second.

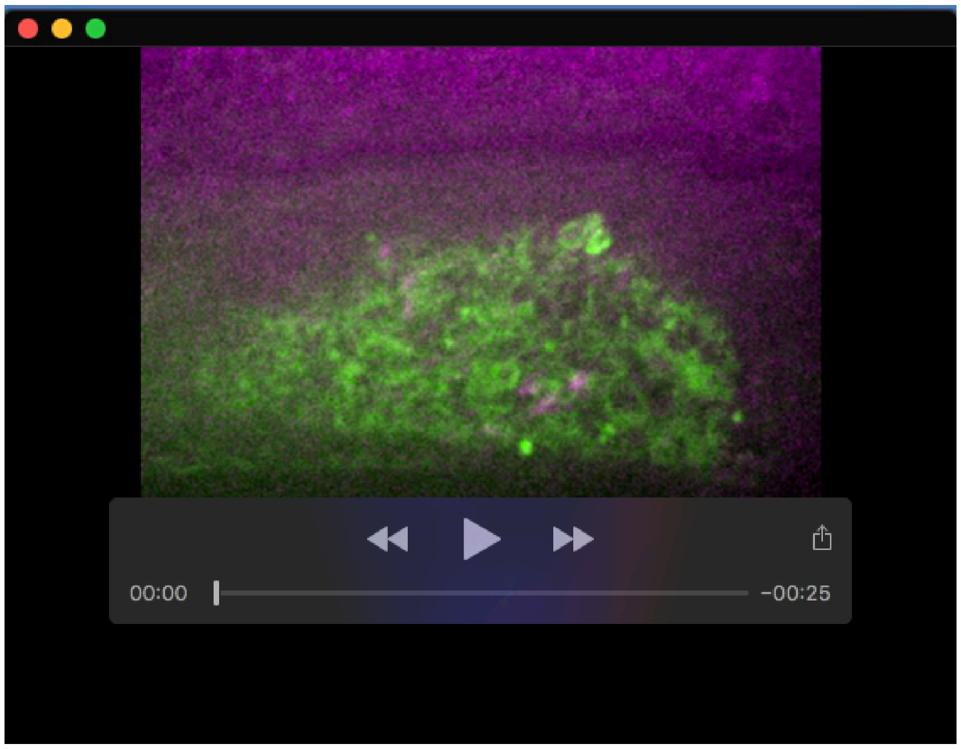

Movie 4. Linear Elements Associate with the Endoplasmic Reticulum

Linear elements (TagRFP::KNL-1, magenta) colocalize with domains of the cortical ER network (SP12::GFP, green), throughout their assembly just prior to NEBD. Single plane images acquired every 5 seconds. Playback speed is 5 frames per second.

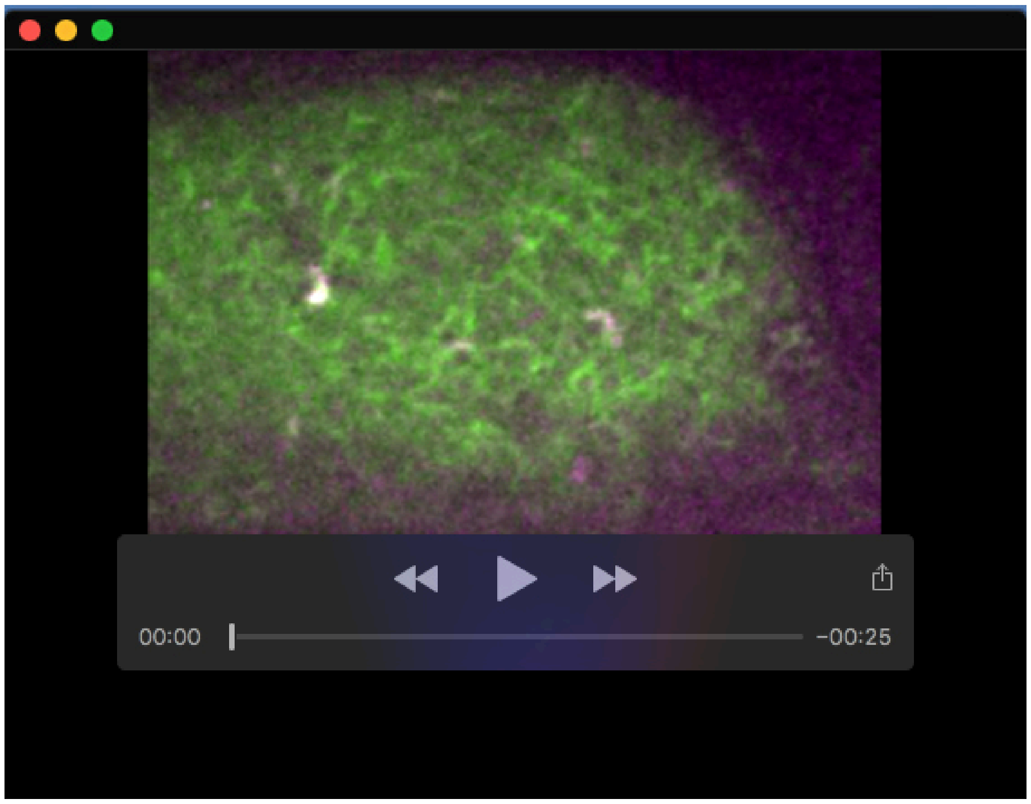

Movie 5. Linear Elements Interact with the Cortical Microtubule Network

Linear elements (TagRFP::KNL-1, magenta) contact microtubules (green) during their assembly before NEBD. Single plane images were acquired every 5 seconds. Playback speed is 10 frames per second.

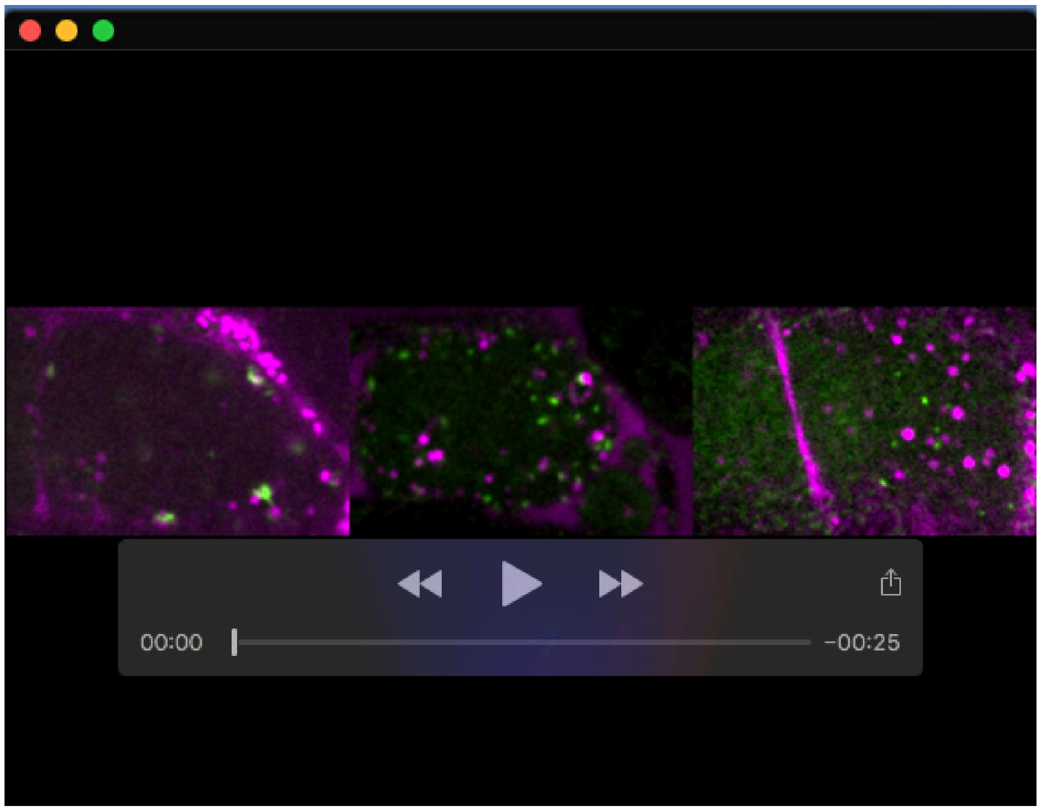

Movie 6. Microtubules and Linear Elements are Required for Vesicle Movement

Linear elements (CZW-1::GFP, green) assemble into clusters with cortical granules (CPG-2::mCherry, magenta) in oocytes of control (left), but not in *tba-2(RNAi)* (middle) or *him-10(RNAi)* (right). Linear elements remain small in both *tba-2(RNAi)* and *him-10(RNAi)* oocytes. In addition, linear elements are relatively immobile in *tba- 2(RNAi)* oocytes, but not *him-10(RNAi)* oocytes. Single plane images were acquired every 5 seconds. Playback speed is 5 frames per second.

Journal of Cell Science • Supplementary information
