# Supplementary material for: Outer kinetochore proteins form linear elements to regulate vesicle transport
Source: J Cell Sci. 2026 May 11;139(9):jcs264478. doi: 10.1242/jcs.264478 (PMC13245919; doi:10.1242/jcs.264478)
Supplement: Supplementary file 1 [file joces-139-264478_review_history.pdf]

Outer kinetochore proteins form linear elements to regulate vesicle transport

Shane J. Kowaleski, Alexis Bridgewater, Cody Saraceno, Miranda Dudek, Federicho Pelisch and Joshua N. Bembenek
DOI: 10.1242/jcs.264478

Editor: Guangshuo Ou

Review timeline

Original submission:	29 September 2025
Editorial decision:	28 October 2025
First revision received:	19 March 2026
Accepted:	5 April 2026

Original submission

First decision letter

MS ID#: jcs.264478

MS TITLE: Outer Kinetochore Proteins form Linear Elements to Regulate Vesicle Transport

AUTHORS: Shane J Kowaleski; Alexis Bridgewater; Cody Saraceno; Miranda Dudek; Federicho Pelisch; Joshua N Bembenek
ARTICLE TYPE: Research Article

Dear Dr Bembenek,

We have now reached a decision on the above manuscript.

As you will see, the reviewers raise a number of substantial criticisms that prevent me from accepting the paper at this stage. They suggest, however, that a revised version might prove acceptable, if you can address their concerns. If you think that you can deal satisfactorily with the criticisms on revision, I would be pleased to see a revised manuscript. We would then return it to the reviewers.

Reviewer 1

General Comments:

The study by Shane J. Kowaleski and Joshua N. Bembenek presents novel insights into the role of outer kinetochore proteins in regulation of vesicle trafficking during oocyte development, specifically during anaphase I in C. elegans oocytes. The authors demonstrate that outer kinetochore proteins form "linear elements" that serve as crucial intermediates in the vesicular transport system. The dynamic formation and growth of linear elements provide evidence for their role in vesicle distribution. Additionally, the findings that microtubules are integral to the assembly and motility of these linear elements further underscore their involvement in cytoskeletal coordination during cell division. The impact of outer kinetochore protein depletion on extracellular matrix formation is an important observation, linking defective vesicle transport to developmental abnormalities. Overall, the study makes novel contribution to the field of cell division and vesicle trafficking.

© 2026. Published by The Company of Biologists under the terms of the Creative Commons Attribution License (https://creativecommons.org/licenses/by/4.0/).

Major comments:

1. The paper suggests that linear elements form punctate sttucture before nuclear envelope breakdown, but more details regarding the molecular cues that trigger their assembly and the exact role of the outer kinetochore proteins in this process would strengthen their findings. Are there any specific post-translational modifications or protein interactions that facilitate their assembly?
2. The authors mentioned that "Linear elements persist in the cortex until mid-anaphase I, and are no longer detectable just before SEP-1 localizes to cortical granules", Why and how do the linear elements disappear? What consequences would arise if the linear elements persisted? The author can appropriately provide explanations for these questions.
3. The relationship between linear elements, microtubules, and the endoplasmic reticulum is intriguing. Does the ER directly contribute to the structural integrity of the linear elements or is it involved in vesicle loading and transport? What is the relationship between ER and the microtubule cytoskeleton?
4. To demonstrate the interaction between the linear elements and cortical granules, in addition to the co-localization analysis, other experimental methods, such as double-molecule fluorescence complementation, could also be employed to support the result.
5. Most of figures are devoid of quantitative and statistical results. For example, the fluorescence co-localization analysis, and the number of linear elements at different stages (before ovulation, oocytes move into the spermatheca, during anaphase I). Meanwhile, the DIC images corresponding to the fluorescence images in Figure 2, 3 and 4 should also be presented to demonstrate the accuracy of the time period selection.
6. The effects of outer kinetochore protein depletion on extracellular matrix formation are highlighted, it would be helpful to provide more insights into the broader cellular consequences of these defects. How do these defects impact overall oocyte viability and developmental outcomes?
7. Depletion of numerous outer kinetochore proteins causes embryo lethality and eggshell defects. What would happen in the dynamics of the linear elements under the condition of knocking down outer kinetochore protein?

Minor comments:

1.The key molecular information in Figure 1A should be clearly indicated.
2.The insets in the bottom of Figure1 B-D should be marked in the corresponding "whole picture" as show in Figure3 A-C.
3.The exact time information should be marked in Figures 1 E and G.
4.In Figure1F, the author should quantify the CZW-1::GFP (green) and SEP-1::mScarlet (magenta) signals on linear elements and chromosomes in multiple embryos over time, and provide error bars and statistical results.
5.The authors observed CZW-1::GFP and CPG-2::mCherry at NEBD after him-10 (RNAi). However, the influence of him-10 (RNAi) on the positioning and morphology of CPG-2 is not mentioned in the text. Why the pattern of CPG-2::mCherry from Figures 5G-I appears to show differences compared with Figures 5A-F?

Reviewer 2

SUMMARY OF THE ADVANCE MADE IN THIS PAPER AND ITS POTENTIAL SIGNIFICANCE TO THE FIELD

This manuscript is an interesting manuscript that describes the non-canonical role of outer kinetochore proteins in C. elegans oocytes, showing that as linear elements, these proteins may participate in an unexpected function related to vesicle transport during meiosis. While numerous kinetochore proteins have previously been observed in these cortical linear structures, their function in oocytes has been unclear; this study suggests they help promote vesicle exocytosis around fertilization.

SUGGESTIONS TO AUTHORS

© 2026. Published by The Company of Biologists under the terms of the Creative Commons Attribution License (https://creativecommons.org/licenses/by/4.0/).

The data are interesting and relevant for the JCS audience, but many of the mechanistic claims—such as direct interaction, dynamic colocalization, or "large cluster" formation—are stronger than is directly supported by the current evidence. The manuscript would benefit from clearer definitions of terms, more careful description of what is shown versus what is inferred, and improvements in figure labeling and quantification where sample sizes are low. Overall, publication is warranted, but the text and presentation should be revised to better support and accurately reflect the data. More detailed comments below.

Figure1:

Line 131: the authors state that "As linear elements assemble into larger structures, they dynamically interact with cortical granules and increasingly colocalize with them (N=6, Figure 2B)". It is unclear what dynamic implies here?

Figure 2. As a non-expert, it is difficult to determine from Figure 2 how the timing of ovulation was established and so a brief explanation would be helpful for readers. The main result here is that CPG-2::mCherry clusters seem to sit right next to the linear elements, but at the moment, this is just shown in a single image. It would make the data stronger if you could quantify or at least describe how often this closeness happens—do all linear elements have these vesicles nearby, or is it just a subset? Even a basic proximity or correlation analysis, or reporting how frequently this is observed, would be important, especially since the sample number is small.

Line 165: "Therefore, linear elements interact with cortical granules while they undergo a complex transport process to become distributed throughout the cortex by anaphase I". or this is purely coincidental…

Figure 3: In this figure, there are some statements about the size of intermediates and how they associate with the ER, but it's not clear what "intermediate size" actually means. It would help if the authors explained how they define or measure these sizes or are the sizes relative to the cell cycle. Also, the linear elements show up where there's stronger ER signal, but saying they "interact" feels too strong without more evidence. It would be better to just describe what's observed and use more careful wording.

Line 180: The authors state that"Therefore, linear elements interact with ER from just before NEBD until anaphase I." Is it possible the linear elements just fall apart by then? Also, the reported N of 2 is really low. If fewer than 5 embryos were examined, it would be good to just state the actual number.

Overall, I found this figure pretty hard to interpret. I wasn't sure what all the different images are supposed to show. Adding clearer labels or more detailed figure legends would make things much easier to follow..

Figure 4: For Figure 4, it is not really clear what the bottom panels are showing. The way they're labeled, it looks like panel G is presenting KNL-1 and microtubule localization, but the legend actually describs it is CPG-2::mCherry instead. Please clarify what each panel actually represents, especially panel H, which is hard to interpret based on the current labels and descriptions.

Line 202: The authors statement "Collectively, these results indicate that linear elements colocalize with microtubules and the ER, and as they grow, they increasingly interact with cortical granule vesicles to form large clusters." Seems a bit strong. The current data show that linear elements are near the ER and might associate with mcirotubules and cortical granules, but there is no evidence for large cluster formation. If the authors want to use that language, they need to clearly define what counts as "large".

Figure 5: For Figure 5, it's again not clear what "smaller" means when describing the linear elements. Please explain how you define or measure this—does it refer to length, signal intensity, or something else? Along those lines, in line 214, where it states "linear elements in tba-2(RNAi) oocytes remained smaller and distributed throughout the cortex until after NEBD (N=12/14, Figure

© 2026. Published by The Company of Biologists under the terms of the Creative Commons Attribution License (https://creativecommons.org/licenses/by/4.0/).

5D-F)", it would be helpful to specify what percent of linear elements don't go to the cortex, and clarify what "smaller" implies

I also found panels J-L a bit confusing—please include a clearer explanation for what the colors represent and what the tracking shows in those images
I am not sure what Figure 5J-L is…..Please include a better explanation for what the colors and the tracking means?

Regarding him-10 RNAi, is the knockdown fully penetrant? Could the leftover puncta be due to some HIM-10 protein still present? If so, it becomes tricky to conclude that HIM-10 isn't needed for motility, since some residual protein might still be there. It would be good for the authors to address this point directly.

Figure6: The permeability data in Figure 6 is convincing. However, I am not sure, based on the images in 6I-N, how to interpret the claim that CPG-2 ends up in the ER in the czw-10 RNAi conditions. It would be a lot easier to tell if you showed the individual panels for the ER marker and CPG-2 separately, instead of just the merged image, the merge seems overwhelmed by the strong CPG-2 signal, making it hard to see any overlap or detail.

First revision

Author response to reviewers' comments

Reviewer 1: General Comments:
The study by Shane J. Kowaleski and Joshua N. Bembenek presents novel insights into the role of outer kinetochore proteins in regulation of vesicle trafficking during oocyte development, specifically during anaphase I in C. elegans oocytes. The authors demonstrate that outer kinetochore proteins form "linear elements" that serve as crucial intermediates in the vesicular transport system. The dynamic formation and growth of linear elements provide evidence for their role in vesicle distribution. Additionally, the findings that microtubules are integral to the assembly and motility of these linear elements further underscore their involvement in cytoskeletal coordination during cell division. The impact of outer kinetochore protein depletion on extracellular matrix formation is an important observation, linking defective vesicle transport to developmental abnormalities. Overall, the study makes novel contribution to the field of cell division and vesicle trafficking.

Major comments:
1. The paper suggests that linear elements form punctate sttucture before nuclear envelope breakdown, but more details regarding the molecular cues that trigger their assembly and the exact role of the outer kinetochore proteins in this process would strengthen their findings. Are there any specific post-translational modifications or protein interactions that facilitate their assembly?

This is an interesting and very broad question. To get initial insight into this question, we tested whether PLK-1 was involved given its localization to linear elements (p.7 168-171). We find that RNAi depletion of PLK-1 inhibits linear element assembly. New data documenting this can be found in Supplemental Figure 2. In our discussion, we also suggest that CDK-1 might regulate linear elements (p.15 430-433) and a recent manuscript from the Bowerman lab has found CDK-1 localized to linear elements (p.15 433-434), which we added to our table of components.

2. The authors mentioned that "Linear elements persist in the cortex until mid-anaphase I, and are no longer detectable just before SEP-1 localizes to cortical granules", Why and how do the linear elements disappear? What consequences would arise if the linear elements persisted? The author can appropriately provide explanations for these questions.

© 2026. Published by The Company of Biologists under the terms of the Creative Commons Attribution License (https://creativecommons.org/licenses/by/4.0/).

This is another very interesting question. The mechanism(s) that regulate linear element disassembly are not currently known. Many kinetochore proteins are known to rapidly shift localization on the spindle during meiosis I, so it might be related to this phenomenon. The only condition we have found that prevents linear element disassembly is APC/C inactivation, as shown in figure S4. However, this condition prevents cell cycle progression generally, obscuring specific effects that might be caused by linear element stabilization. Further insights into this question require identifying conditions that stabilize linear elements independently of cell cycle progression, which we feel is beyond the scope of this manuscript.

3. The relationship between linear elements, microtubules, and the endoplasmic reticulum is intriguing. Does the ER directly contribute to the structural integrity of the linear elements or is it involved in vesicle loading and transport? What is the relationship between ER and the microtubule cytoskeleton?

We agree that the role of the ER is intriguing. Given that ZW10 has been found in a complex of proteins that function at ER exit sites, we hypothesize that this might be a point of contact between linear elements and membranes. To gain insight into this, we found that *czw-1(RNAi)* inhibits cortical granule formation (Figure 6), which occurs during the extended prophase arrest preceding linear element formation. This indicates that ZW10 has an additional role earlier in vesicle biogenesis in addition to its role(s) at linear elements. Previously, Kimura et al. demonstrated that ER morphology affects microtubule-dependent cytoplasmic streaming, which we cited. We also find that depletion of microtubules significantly impacts ER morphology and cortical displacement throughout meiosis I, as shown in Figure S3. We define multiple stages of vesicle formation, transport and eventual exocytosis. Long term depletions by RNAi do not allow us to evaluate the role of the ER and microtubules at each step but rather observe chronic effects. The function of ER and microtubules likely changes during each stage of this process and will require future studies to fully understand.

4. To demonstrate the interaction between the linear elements and cortical granules, in addition to the co-localization analysis, other experimental methods, such as double-molecule fluorescence complementation, could also be employed to support the result.

While we agree that this experiment would indeed be very helpful, we do not have the tools to perform this experiment. We also do not currently know the molecular interaction between kinetochore proteins and vesicles, so we feel that this currently falls outside the scope of the current manuscript.

5. Most of figures are devoid of quantitative and statistical results. For example, the fluorescence co-localization analysis, and the number of linear elements at different stages (before ovulation, oocytes move into the spermatheca, during anaphase I). Meanwhile, the DIC images corresponding to the fluorescence images in Figure 2, 3 and 4 should also be presented to demonstrate the accuracy of the time period selection.

We have added several important quantifications, including linear element size (p.6-7 145-158) and the intensity (p.6 134-139) of CZW-1 and SEP-1 at different stages (puncta, intermediate, long and condensed) that we defined in control during their formation around the time of NEBD (Figure 1F). We also measured the lengths of linear elements in *tba-2(RNAi)* and *him-10(RNAi)* at their longest point prior to ovulation and found that they were significantly shorter than control (Figure 5J, p.10 277-279 and p.11 312-314). We also quantified the “vesicle clustering” phenomena by measuring the distance between each linear element and the nearest vesicle. We observed a significant decrease in median distance for “condensed” stage linear elements compared to earlier stages, confirming the increased colocalization of linear elements with vesicles over time (Figure 2H). We also found that clustering is significantly inhibited in *tba-2(RNAi)* and *him-10(RNAi),* as shown in (Figure 5O, p.10 282-286 and p.11 314-318).

Finally, we extensively examined the timeline of events including linear element formation and growth relative to NEBD and ovulation, as shown in Figure S3. Our timeline maps numerous additional events over the original timeline published by McCarter et al., (1999) which was done with DIC imaging. We found that ovulation, the point where the oocyte moves into the spermatheca, is a very reliable timepoint relative to linear element formation and can easily be observed regardless of which plane we were imaging. Therefore, all events are presented relative

© 2026. Published by The Company of Biologists under the terms of the Creative Commons Attribution License (https://creativecommons.org/licenses/by/4.0/).

to ovulation rather than using DIC to indicate stage, which would be less reliable than accurate timeline information. Also, streaming occurs after the oocyte moves into the spermatheca, and anaphase occurs when the zygote moves into the uterus. Therefore, all of these markers have been systematically used to select ideal examples at the correct stage instead of using DIC images.

6. The effects of outer kinetochore protein depletion on extracellular matrix formation are highlighted, it would be helpful to provide more insights into the broader cellular consequences of these defects. How do these defects impact overall oocyte viability and developmental outcomes?

Formation of the extracellular matrix is critical for oocytes to protect against osmotic and mechanical perturbations for viability. Furthermore, this process is linked to the block to polyspermy in many species. We highlight these issues in the introduction. (p.3 75-78).

7. Depletion of numerous outer kinetochore proteins causes embryo lethality and eggshell defects. What would happen in the dynamics of the linear elements under the condition of knocking down outer kinetochore protein?

We believe this might depend on exactly which kinetochore proteins are knocked down. We found that linear elements do not form properly in *him-10(RNAi)* embryos, as shown in Figure 5. Interestingly, CZW-1 labeled puncta are still motile after *him-10(RNAi)*, suggesting they are still capable of microtubule-based movements. In contrast, depletion of microtubules inhibits linear element movements and inhibits their formation. So different kinetochore proteins may have different roles in formation and motility.

Minor comments:
1.The key molecular information in Figure 1A should be clearly indicated.

This has been added.

2.The insets in the bottom of Figure1 B-D should be marked in the corresponding "whole picture" as show in Figure3 A-C.

The insets for figure 1B-1D are single plane images that we chose that best represent the shape of linear elements and the colocalization between CZW-1 and SEP-1 at different stages. They are not crops from the whole cell images above, which is why we did not include a marking for the crop region as we did in figure 3A-3C. We clarified this in the figure legend (p.18 489-491).

3.The exact time information should be marked in Figures 1 E and G.

This has been fixed.

4.In Figure1F, the author should quantify the CZW-1::GFP (green) and SEP-1::mScarlet (magenta) signals on linear elements and chromosomes in multiple embryos over time, and provide error bars and statistical results.

This has been added to Figure 1.

5.The authors observed CZW-1::GFP and CPG-2::mCherry at NEBD after him-10 (RNAi). However, the influence of him-10 (RNAi) on the positioning and morphology of CPG-2 is not mentioned in the text. Why the pattern of CPG-2::mCherry from Figures 5G-I appears to show differences compared with Figures 5A-F?

In the results section, we say "Linear element clustering with cortical granules was also inhibited before ovulation (Figure 5G-I, 5N, Movie S6), with the average median distance to nearest vesicle being 0.929±0.14 μm (N=12), approximately four times greater than that of control (0.21±0.074 μm, N=16, Figure 5O, p=0.044)." (p. 11 314-318). We did not notice any changes in vesicle morphology, which would be difficult to characterize with the resolution of our images.

© 2026. Published by The Company of Biologists under the terms of the Creative Commons Attribution License (https://creativecommons.org/licenses/by/4.0/).

Reviewer 2: SUMMARY OF THE ADVANCE MADE IN THIS PAPER AND ITS POTENTIAL SIGNIFICANCE TO THE FIELD

This manuscript is an interesting manuscript that describes the non-canonical role of outer kinetochore proteins in C. elegans oocytes, showing that as linear elements, these proteins may participate in an unexpected function related to vesicle transport during meiosis. While numerous kinetochore proteins have previously been observed in these cortical linear structures, their function in oocytes has been unclear; this study suggests they help promote vesicle exocytosis around fertilization.

SUGGESTIONS TO AUTHORS

The data are interesting and relevant for the JCS audience, but many of the mechanistic claims—such as direct interaction, dynamic colocalization, or "large cluster" formation—are stronger than is directly supported by the current evidence. The manuscript would benefit from clearer definitions of terms, more careful description of what is shown versus what is inferred, and improvements in figure labeling and quantification where sample sizes are low. Overall, publication is warranted, but the text and presentation should be revised to better support and accurately reflect the data. More detailed comments below.

Figure1: Line 131: the authors state that "As linear elements assemble into larger structures, they dynamically interact with cortical granules and increasingly colocalize with them (N=6, Figure 2B)". It is unclear what dynamic implies here?

This language is trying to describe how linear elements appear to explore the cortex and interact with vesicles over time. In our movies you can see them moving constantly, exploring the cortical cytoplasm. As they move they seem to find vesicles and then cluster with them as they grow bigger. The process appears different than how a kinetochore is formed on the surface of a chromosome and then is captured by spindle microtubules and then pulled towards the spindle poles. We are also trying to use careful wording to avoid implying definitive mechanistic descriptions. To clarify this, we revised the statement to: "As linear elements assemble into larger structures, they dynamically move in the cortical cytoplasm and increasingly colocalize with cortical granules over time (N=6, Figure 2A-C, 2H, Movie S2)." (p. 7 182-184).

Figure 2. As a non-expert, it is difficult to determine from Figure 2 how the timing of ovulation was established and so a brief explanation would be helpful for readers. The main result here is that CPG-2::mCherry clusters seem to sit right next to the linear elements, but at the moment, this is just shown in a single image. It would make the data stronger if you could quantify or at least describe how often this closeness happens—do all linear elements have these vesicles nearby, or is it just a subset? Even a basic proximity or correlation analysis, or reporting how frequently this is observed, would be important, especially since the sample number is small.

A statement describing how we define ovulation has been added to the results (p.6 139-140). We have now quantified several aspects of the process, including a quantification of the "vesicle clustering" phenomena by measuring the distance between each linear element and the nearest vesicle. We observed a significant decrease in median distance for "condensed" stage linear elements compared to earlier stages, confirming the increased colocalization of linear elements with vesicles over time (Figure 2H). We also found that clustering is significantly inhibited in *tba-2(RNAi)* and *him-10(RNAi),* as shown in (Figure 5O, p.10 282-286 and p.11 314-318).

Line 165: "Therefore, linear elements interact with cortical granules while they undergo a complex transport process to become distributed throughout the cortex by anaphase I". or this is purely coincidental…

We agree that colocalization alone does not demonstrate a direct interaction. In later sections of the manuscript, we show that inhibition of microtubules or kinetochore proteins affects linear element size and vesicle clustering, supporting a functional relationship between these structures. To avoid implying a mechanistic interaction in this section, we have revised the sentence to: "Together, these observations show that linear elements colocalize with cortical

© 2026. Published by The Company of Biologists under the terms of the Creative Commons Attribution License (https://creativecommons.org/licenses/by/4.0/).

granules around the time of NEBD during a complex transport process that distributes cortical granules throughout the cortex by anaphase I." (p.8 218-221).

Figure 3: In this figure, there are some statements about the size of intermediates and how they associate with the ER, but it's not clear what "intermediate size" actually means. It would help if the authors explained how they define or measure these sizes or are the sizes relative to the cell cycle. Also, the linear elements show up where there's stronger ER signal, but saying they "interact" feels too strong without more evidence. It would be better to just describe what's observed and use more careful wording.

We have clarified what is meant by "intermediate size" and quantified linear element length over time, defined the progression of stages (puncta, intermediate, long, condensed) based on these measurements, with the corresponding timing indicated relative to ovulation (Figure 1). Also, we revised our descriptions to more carefully reflect our observations of the ER and linear elements (p.9 227-237). We also added additional nuclear plane images to make it easier to appreciate how linear elements form and are embedded within the cortical ER network and around the nuclear envelope (Figure 3A-C).

Line 180: The authors state that"Therefore, linear elements interact with ER from just before NEBD until anaphase I." Is it possible the linear elements just fall apart by then? Also, the reported N of 2 is really low. If fewer than 5 embryos were examined, it would be good to just state the actual number.

Yes, the linear elements as labeled with CZW-1 disappear right before separase accumulates on vesicles in anaphase, which we show in Figure 1H. We increased our N of this stage.

Overall, I found this figure pretty hard to interpret. I wasn't sure what all the different images are supposed to show. Adding clearer labels or more detailed figure legends would make things much easier to follow..

We added nuclear plane images to Figure 3 to help clarify the localization pattern, additional labels, and improved the legend to address this concern. (p.19 531-535).

Figure 4: For Figure 4, it is not really clear what the bottom panels are showing. The way they're labeled, it looks like panel G is presenting KNL-1 and microtubule localization, but the legend actually describs it is CPG-2::mCherry instead. Please clarify what each panel actually represents, especially panel H, which is hard to interpret based on the current labels and descriptions.

We have added labels to Figure 4 to clarify the fluorescent labels in the image. A scale bar with timestamps has been added to the color projection to clarify the timescale that the colors in the projection represent. Further detail on this panel has been added to the figure legend. (p.20 554-567).

Line 202: The authors statement "Collectively, these results indicate that linear elements colocalize with microtubules and the ER, and as they grow, they increasingly interact with cortical granule vesicles to form large clusters." Seems a bit strong. The current data show that linear elements are near the ER and might associate with mcirotubules and cortical granules, but there is no evidence for large cluster formation. If the authors want to use that language, they need to clearly define what counts as "large".

We agree that the term "large" was not clearly defined and could be misleading. Our intention was simply to indicate that multiple cortical granules are present within each cluster. We have revised this statement to read: "Collectively, these results indicate that linear elements colocalize with microtubules and the ER, and form clusters with cortical granules as they assemble prior to ovulation." (p.10 265-267).

Figure 5: For Figure 5, it's again not clear what "smaller" means when describing the linear elements. Please explain how you define or measure this—does it refer to length, signal intensity, or something else? Along those lines, in line 214, where it states "linear elements in tba-2(RNAi) oocytes remained smaller and distributed throughout the cortex until after NEBD (N=12/14, Figure

© 2026. Published by The Company of Biologists under the terms of the Creative Commons Attribution License (https://creativecommons.org/licenses/by/4.0/).

5D-F)", it would be helpful to specify what percent of linear elements don't go to the cortex, and clarify what "smaller" implies

To address this, we measured the length of linear elements from single plane images in empty vector control, *tba-2(RNAi)* and *him-10(RNAi)* and found that they are significantly shorter in mutant conditions. Details of this quantification have been added to the results and methods sections, and a plot of the quantification has been added to Figure 5 (Figure 5J, p.10 277-282 and p.11 312-314).

Regarding distribution relative to the cortex, we have removed this description. We show that microtubule disruption alters ER organization in *tba-2(RNAi)* oocytes (Figure S3). Since linear elements remain closely associated with ER-rich regions, we do not currently have sufficient evidence to interpret whether these positional differences reflect a specific defect in linear element behavior or a secondary consequence of global changes in cellular organization.

I also found panels J-L a bit confusing—please include a clearer explanation for what the colors represent and what the tracking shows in those images
I am not sure what Figure 5J-L is…..Please include a better explanation for what the colors and the tracking means?

We have added labels to clarify the time represented by the scalebar within this figure. A statement describing what the colors represent has been added to the figure legend to further clarify this. (p.21 585-590).

Regarding him-10 RNAi, is the knockdown fully penetrant? Could the leftover puncta be due to some HIM-10 protein still present? If so, it becomes tricky to conclude that HIM-10 isn't needed for motility, since some residual protein might still be there. It would be good for the authors to address this point directly.

We agree that it is unlikely that all HIM-10 protein has been depleted from the cell. When we measured the length of linear elements in *him-10(RNAi)* embryos, we observed a range of linear element size in different oocytes, suggesting differences in severity of knockdown. (p.11 310-312). We note that *him-10(RNAi)* does not inhibit motility of linear elements regardless of size. We have revised our statements: "Interestingly, linear element puncta in *him-10(RNAi)* oocytes are still motile and move dynamically in the cortex (Figure 5L). Therefore, Nuf2$^{him\text{-}10}$ is required for linear element assembly and cortical granule clustering but may not be required for motility." (p.11 318-321)

Figure6: The permeability data in Figure 6 is convincing. However, I am not sure, based on the images in 6I-N, how to interpret the claim that CPG-2 ends up in the ER in the czw-10 RNAi conditions. It would be a lot easier to tell if you showed the individual panels for the ER marker and CPG-2 separately, instead of just the merged image, the merge seems overwhelmed by the strong CPG-2 signal, making it hard to see any overlap or detail.

We added single channel insets beneath the images to clarify this overlap in the severe *czw-1(RNAi)* condition, and labeled the region where the crop was taken from on the image above it. (Figure 6I-K).

<u>Second decision letter</u>

MS ID#: jcs.264478R1

MS Title: Outer Kinetochore Proteins form Linear Elements to Regulate Vesicle Transport

Authors: Shane J Kowaleski; Alexis Bridgewater; Cody Saraceno; Miranda Dudek; Federicho Pelisch; Joshua N Bembenek
Article Type: Research Article

© 2026. Published by The Company of Biologists under the terms of the Creative Commons Attribution License (https://creativecommons.org/licenses/by/4.0/).

Dear Dr Bembenek,

I am happy to tell you that your manuscript has been accepted for publication in Journal of Cell Science, pending standard publication integrity checks.

© 2026. Published by The Company of Biologists under the terms of the Creative Commons Attribution License (https://creativecommons.org/licenses/by/4.0/).
